# A tumor microenvironment-responsive poly(amidoamine) dendrimer nanoplatform for hypoxia-responsive chemo/chemodynamic therapy

**DOI:** 10.1186/s12951-022-01247-6

**Published:** 2022-01-21

**Authors:** Yingchao Hao, Yue Gao, Yu Fan, Changchang Zhang, Mengsi Zhan, Xueyan Cao, Xiangyang Shi, Rui Guo

**Affiliations:** State Key Laboratory for Modification of Chemical Fibers and Polymer Materials, Shanghai Engineering Research Center of Nano-Biomaterials and Regenerative Medicine, College of Chemistry, Chemical Engineering and Biotechnology, Donghua University, Shanghai, 201620 China

**Keywords:** Tumor microenvironment-responsive, Chemodynamic therapy (CDT), Tirapazamine (TPZ), Synergistic therapy, PAMAM dendrimer

## Abstract

**Background:**

Chemodynamic therapy is a promising cancer treatment with specific therapeutic effect at tumor sites, as toxic hydroxyl radical (·OH) could only be generated by Fenton or Fenton-like reaction in the tumor microenvironment (TME) with low pH and high level of endogenous hydrogen peroxide. However, the low concentration of catalytic metal ions, excessive glutathione (GSH) and aggressive hypoxia at tumor site seriously restrict the curative outcomes of conventional chemodynamic therapy.

**Results:**

In this study, polyethylene glycol-phenylboronic acid (PEG-PBA)-modified generation 5 (G5) poly(amidoamine) (PAMAM) dendrimers were synthesized as a targeted nanocarrier to chelate Cu(II) and then encapsulate hypoxia-sensitive drug tirapazamine (TPZ) by the formation of hydrophobic Cu(II)/TPZ complex for hypoxia-enhanced chemo/chemodynamic therapy. The formed G5.NHAc-PEG-PBA@Cu(II)/TPZ (GPPCT) nanoplatform has good stability and hemocompatibility, and could release Cu(II) ions and TPZ quickly in weakly acidic tumor sites via pH-sensitive dissociation of Cu(II)/TPZ. In vitro experiments showed that the GPPCT nanoplatforms can efficiently target murine breast cancer cells (4T1) cells overexpressing sialic acid residues, and show a significantly enhanced inhibitory effect on hypoxic cells by the activation of TPZ. The excessive GSH in tumors could be depleted by the reduction of Cu(II) to Cu(I), and abundant of toxic ·OH would be generated in tumor cells by Fenton reaction for chemodynamic therapy. In vivo experiments demonstrated that the GPPCT nanoplatform could specifically accumulate at tumors, effectively inhibit the growth and metastasis of tumors by the combination of CDT and chemotherapy, and be metabolized with no systemic toxicity.

**Conclusions:**

The targeted GPPCT nanoplatform may represent an effective model for the synergistic inhibition of different tumor types by hypoxia-enhanced chemo/chemodynamic therapy.

**Graphical Abstract:**

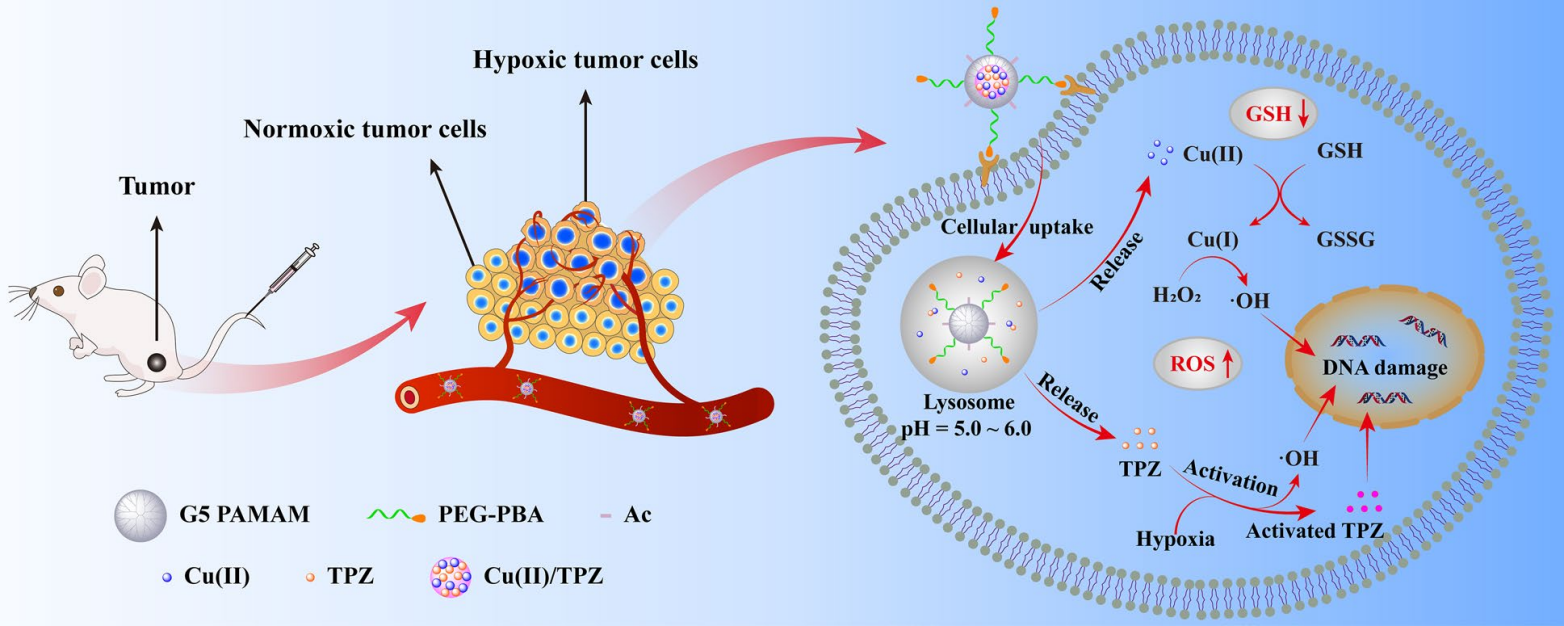

**Supplementary Information:**

The online version contains supplementary material available at 10.1186/s12951-022-01247-6.

## Introduction

Tumor microenvironment (TME) is characterized by weak acidity, high concentration of hydrogen peroxide (H_2_O_2_), excessive glutathione (GSH), and hypoxia [1–3]. It has been well proved that TME not only plays an important role in the occurrence, development and metastasis of tumors [4, 5], but also induces resistance to conventional anticancer treatments such as radiotherapy and chemotherapy, resulting in poor prognosis [5, 6]. Nevertheless, TME could also be exploited to realize certain specific cancer treatments owing to its notable difference to normal tissues [7]. In addition, normalization of TME may in turn improve tumor therapy outcome [4]. Thereby a large number of TME-responsive cancer treatment strategies have been developed in recent years [8–10], including chemodynamic therapy (CDT) [11–13] and hypoxic-sensitive chemotherapy [14, 15].

CDT is known as an emerging ROS (reactive oxygen species)-mediated cancer treatment based on in situ Fenton reaction at tumor sites [3]. In a typical CDT, transition metal ions, such as Fe(II) [16, 17], Cu(I) [11–13] and Mn(II) [18], are introduced to tumor sites, and highly toxic hydroxyl radicals (·OH) are generated by catalyzing the excess endogenous H_2_O_2_ at TME via Fenton or Fenton-like reaction [2]. CDT could specifically lead to tumor cell death and show neglectable toxicity to normal tissues due to their low H_2_O_2_ level and neutral pH. Various nanoplatforms have been developed to deliver catalytic ions to tumors [19], such as amorphous iron nanoparticles (NPs) [16], ferrous-cysteine-phosphotungstate NPs [17], self-assembled copper-amino acid NPs [11], copper peroxide nanodots [12], nanocatalytic Cu-GSSG NPs [13], Mn-Cu bimetallic nanocomplexes [18], etc. Among them, Cu(II) has attracted increasing attention as the CDT catalyst, since the elevated level of GSH in TME could be depleted by the reduction of Cu(II) to Cu(I) [18, 20], and Cu(I) possesses a 160 times higher catalytic efficiency than Fe(II) under the weak acidic condition, leading to effective killing of tumor cells [21–23]. For instance, Ma et al. [11] synthesized copper-amino acid nanoparticles (Cu-Cys NPs) and demonstrated the excessive GSH and H_2_O_2_ in TME may activate and reinforce the chemodynamic therapeutic effect. Fan et al. [24] synthesized pyridine (Pyr) modified generation 5 (G5) poly(amidoamine) (PAMAM) dendrimer to complex Cu(II), and proved that the formed hybrid nanoplatform G5.NHAc-Pyr/Cu(II) could display an enhanced inhibition of tumor proliferation and metastasis with the combination of radiotherapy. Despite various successes reported on CDT, the actual efficacy of CDT alone is often limited due to the lack of adequate concentration of catalytic ions at tumors and the low Fenton reaction catalytic activity under hypoxia condition [25–27], and hence combination therapy will be beneficial complement to cancer treatment.

Hypoxia is a common and unique feature of malignant solid tumors [1, 2], which may promote the tumor metastatic progression and weaken the traditional chemotherapeutic effects [6]. Among various drugs, hypoxia-activated prodrugs are of particular interest [28], because they can be converted to potent anticancer drugs through specific reductase in hypoxic cells [25, 29] and effectively kill hypoxic tumor cells within malignant solid tumors [27, 30]. It is reasonable to speculate that the combination of hypoxia-sensitive chemotherapy and CDT may enhance the antitumor efficacy to hypoxia tumors and result in the cooperative enhancement in tumor inhibition [31, 32]. As the lead compound in benzotriazine-di-N-oxide class of hypoxic cytotoxins, tirapazamine (TPZ) may undergo two different types of breakage reactions in hypoxic tumor cells to produce ·OH and benzotriazinyl (BTZ) radical, inducing DNA strand breakage and topoisomerase II poisoning (Additional file 1: Fig. S1) [14, 26]. In recent work, Wang et al. [14] synthesized ferrocene-containing polymersome nanoreactors to load TPZ and glucose oxidase, and demonstrated that TPZ could be activated at hypoxic tumors and synergistically inhibit tumors with ·OH generated through Fenton reaction by ferrocene. Therefore, it is highly desirable and challenging to integrate CDT and hypoxia-sensitive chemotherapy into one nanoplatform, which can specifically deliver CDT catalyst and hypoxia-sensitive drugs to tumor sites, promote drug release and uptake by tumor cells, and exert both therapies specifically within tumors to obtain synergistic therapeutic effect.

**Scheme 1:**
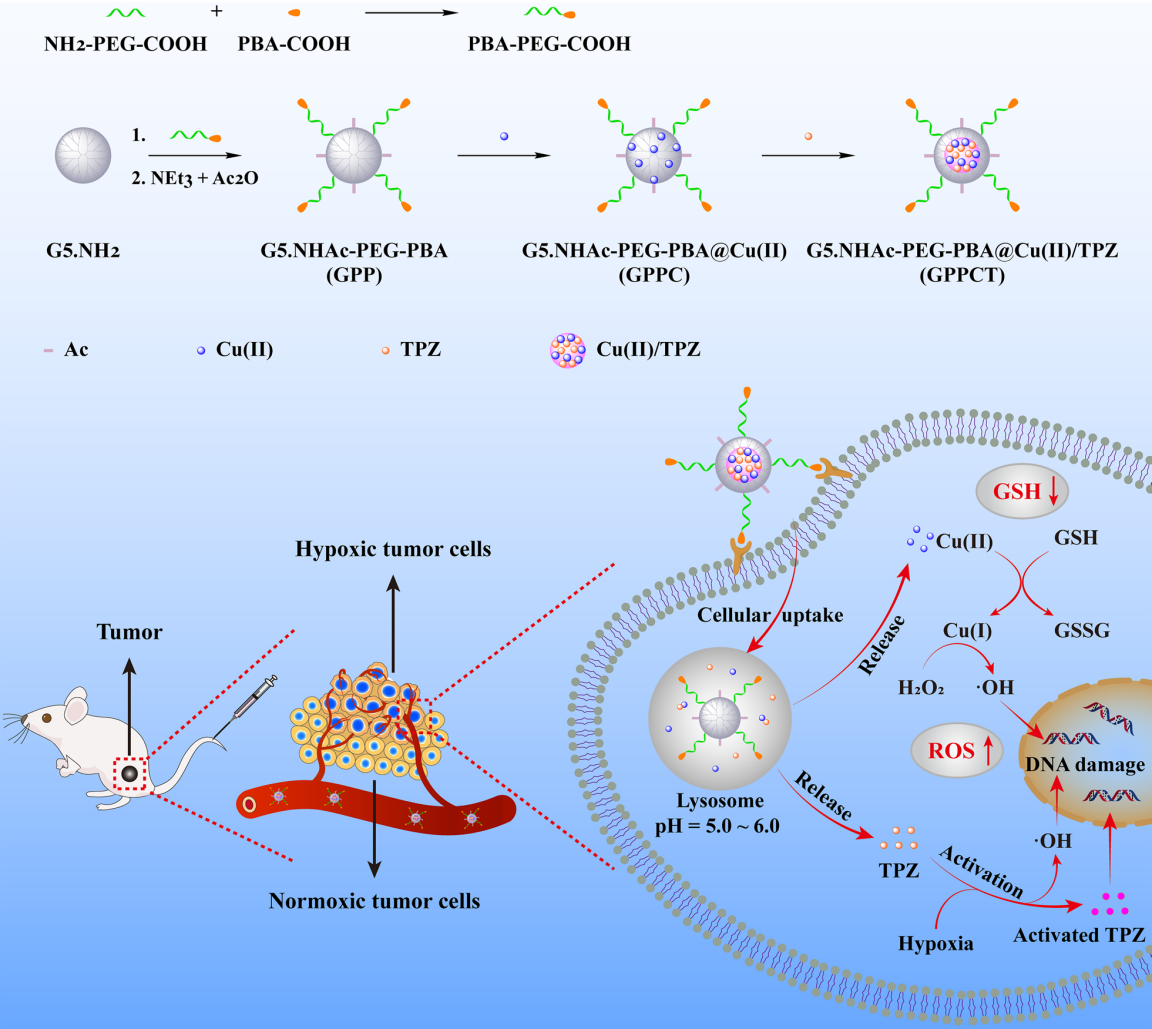
Schematic illustration of the synthesis of G5.NHAc-PEG-PBA@Cu(II)/TPZ (GPPCT) and the corresponding therapeutic mechanisms against tumor cells

Herein, as shown in Scheme 1, we synthesized a targeted nanocarrier based on polyethylene glycol-phenylboronic acid (PEG-PBA) modified PAMAM dendrimers to chelate copper ions firstly, and then load TPZ in dendrimers via the formation of hydrophobic Cu(II)/TPZ complexes. Due to the specific interaction between PBA and the sialic acid residues overexpressed on tumor cells, the obtained G5.NHAc-PEG-PBA@Cu(II)/TPZ (GPPCT) nanoplatform can be preferably enriched in tumor site, and rapidly release Cu(II) and TPZ at the weakly acidic TME by the pH-responsive dissociation of Cu(II)/TPZ complexes. Then Cu(II) may be reduced to Cu(I) by the excessive GSH in tumor cells, and produce toxic ·OH by Fenton-like reaction with endogenous H_2_O_2_. At the same time, the released TPZ may produce BTZ radical and ·OH in tumor hypoxic region, inducing the synergistic effect of chemotherapy and chemodynamic therapy. In addition, GPPCT nanoplatform could decrease the tumor metastasis and show low systemic toxicity. Thus, the designed TME-responsive GPPCT nanoplatform could specifically and effectively eliminate tumors by the synergistic chemodynamic therapy and hypoxia-enhanced chemotherapy, representing an efficient and safe strategy for tumor therapy.

## Results and discussion

### Synthesis and characterization of G5.NHAc-PEG-PBA@Cu(II)/TPZ (GPPCT)

**Fig. 1:**
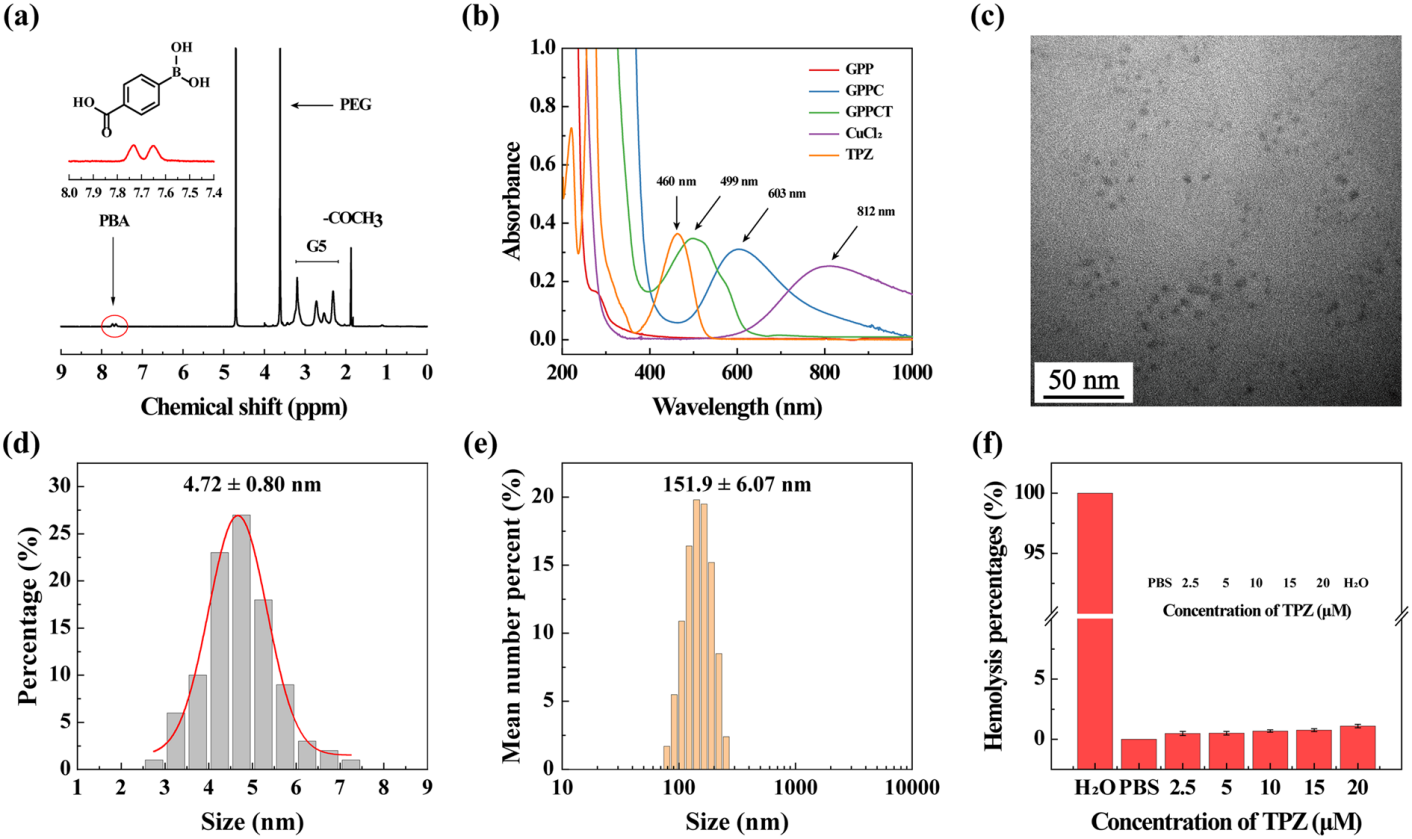
**a** ^1^H NMR spectra of GPP. **b** UV–V is spectra of GPP, GPPC, GPPCT, CuCl_2_ and TPZ. **c** TEM image and **d** size distribution histogram of GPPCT. **e** Hydrodynamic size distribution histogram of GPPCT. **f** Hematolysis rate and photographs of blood samples treated with PBS or GPPCT at different TPZ concentrations

In this study, G5.NHAc-PEG-PBA (GPP) nanocarriers targeting cancer cells overexpressing sialic acid residues were firstly synthesized by conjugating 4-carboxylphenylboronic acid (PBA) onto G5 dendrimer with a PEG chain spacer and then acetylation of remaining amino groups on surface. The structure of GPP was confirmed by ^1^H NMR spectra in Fig. 1a. The presence of the characteristic peak of PBA at 7.60–7.78 ppm, PEG at 3.61 ppm, and acetyl groups at 1.88 ppm indicated the successful synthesis of GPP. By integral calculation, each GPP possessed about 6.8 PEG, 2.9 PBA and 73.3 acetyl groups (Additional file 1: Figs. S2a, b). As a non-targeted control, G5.NHAc-*m*PEG (G*m*P) (Additional file 1: Fig. S3) with an average of 7.0 *m*PEG and 74.3 acetyl groups was synthesized by conjugating *m*PEG on the surface of G5 (Additional file 1: Fig. S2c, d). Then, Cu(II) was complexed into the dendrimers by the tertiary amine groups, and TPZ molecules were loaded via the formation of hydrophobic Cu(II)/TPZ nanocomplexes to yield G5.NHAc-PEG-PBA@Cu(II)/TPZ (GPPCT) and G5.NHAc-*m*PEG@Cu(II)/TPZ (G*m*PCT). UV–Vis spectra in Fig. 1b and Additional file 1: Fig. S4 demonstrated the stepwise loading of drugs in GPP and G*m*P. After the chelating Cu(II), GPPC displayed an obvious absorption peak at 603 nm, indicating that Cu(II) had been successfully loaded into GPP. And the peak at 499 nm in GPPCT proved the encapsulation of TPZ and the formation of Cu(II)/TPZ complexes in nanoplatforms. The loading content (LC) and entrapment efficiency (EE) of TPZ in GPPCT were calculated to be 2.26% and 65.03%, and those for G*m*PCT were 2.38% and 66.67%, respectively (Additional file 1: Table S1). In addition, the amount of Cu(II) in nanocomplexes was also measured by ICP-OES, and the average number of Cu(II) in each GPPCT and G*m*PCT was calculated to be 17.69 and 18.07 per dendrimer, respectively. Finally, the morphologies of GPPCT and G*m*PCT were evaluated by TEM in Fig. 1c and Additional file 1: Fig. S5a. Both nanoplatforms showed regular spherical structure with good mono-dispersity, and the mean size of Cu(II)/TPZ complexes in GPPCT and G*m*PCT were about 4.72 ± 0.80 nm and 4.74 ± 0.87 nm (Fig. 1d and Additional file 1: Fig. S5b). Both nanoplatforms had a similar mean hydrodynamic diameter of 150 nm (Fig. 1e, Additional file 1: Table S2 and Fig. S6), and kept good stability in water, PBS and DMEM media within seven days (Additional file 1: Fig. S7). As a result, targeted GPPCT and non-targeted G*m*PCT with similar size and drug content had been successfully synthesized for further comparison. To ensure the safe application of nanoplatforms in vivo, the hemolysis rate of GPPCT ([TPZ] = 2.5, 5, 10, 15, 20 μM) was evaluated in Fig. 1f. The red blood cells in the positive control group ruptured and the solution turned red, while the hemolysis rate of GPPCT nanoplatform was less than 5%, indicating their good blood compatibility. Therefore, Cu(II) and TPZ loaded GPPCT nanoplatform with good colloidal stability and hemocompatibility were successfully synthesized for biomedical applications.

**Fig. 2:**
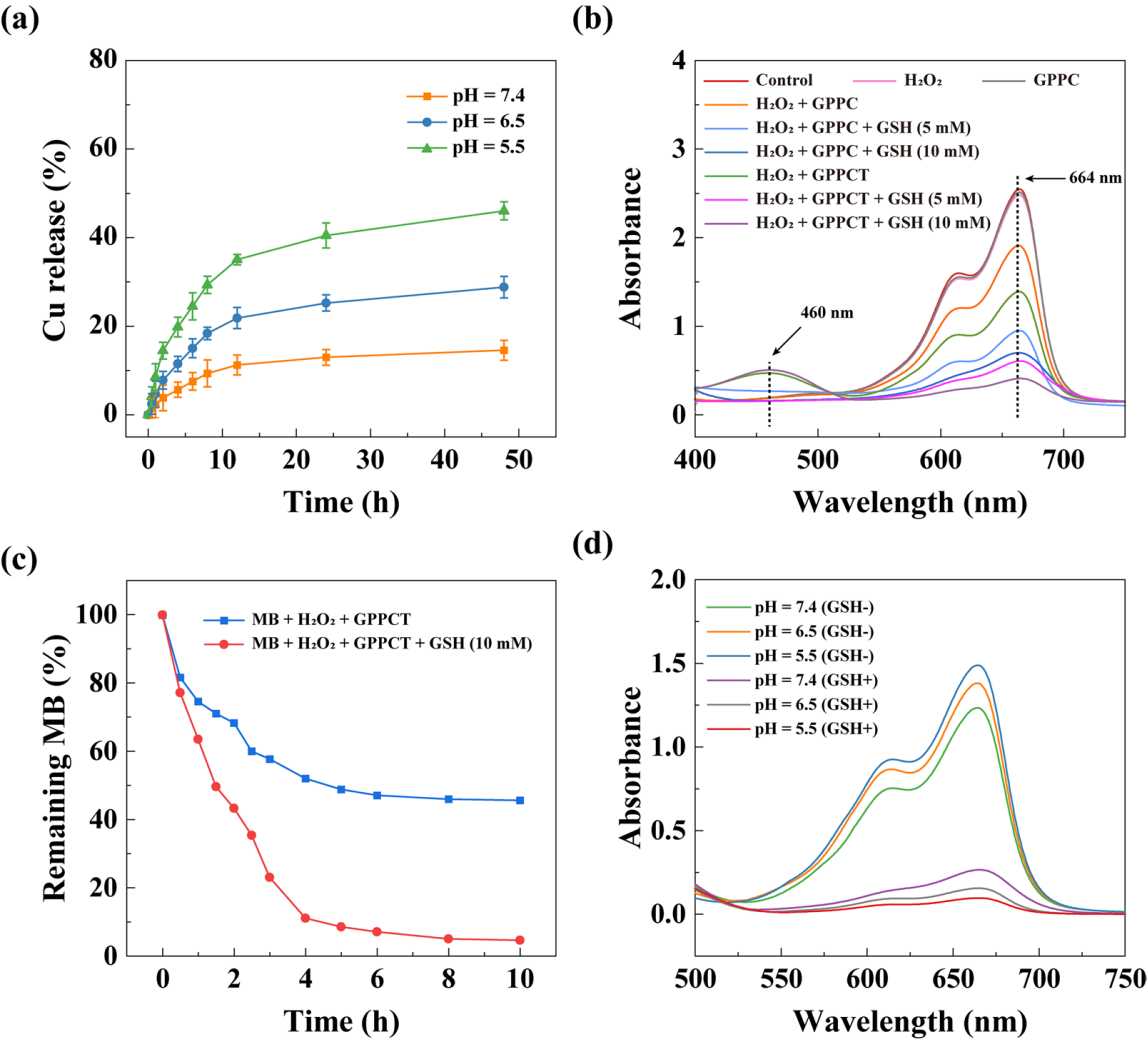
**a** Accumulative release of Cu(II) from GPPCT in buffer solutions at different pH of 5.5, 6.5 and 7.4 ([GPPCT] = 1 mg/mL). **b** UV–Vis spectra of MB solutions treated with H_2_O_2_ and GPPC or GPPCT with or without the presence of GSH for 4 h. **c** Degradation rate of MB treated with H_2_O_2_ and GPPCT with or without the presence of GSH within 10 h. **d** UV–Vis spectra of MB treated with H_2_O_2_ and GPPCT with or without the presence of GSH at different pH of 5.5, 6.5 and 7.4. For **b**–**d**, the mixtures were kept under 37 °C, MB = 10 μg/mL, [GSH] = 5 mM or 10 mM, [H_2_O_2_] = 10 mM, [TPZ] = 200 μM, and [Cu(II)] = 616 μM)

The drug release profiles of GPPCT at pH 7.4, 6.5 and 5.5, which is to mimic the physiological environment, TME and weak acidic environment in lysosome respectively, were studied by ICP-OES for Cu and UV–Vis spectroscopy for TPZ. As shown in Fig. 2a, the amount of Cu released at pH 5.5 within 48 h was about 3.15 times higher than that released at pH 7.4 (46.05% vs 14.56%). A similar pH-sensitive release profile of TPZ could also be observed in Additional file 1: Fig. S8a, and a significantly higher amount of TPZ was released under weak acidic condition than in physiological situation. The pH-sensitive release property of GPPCT should be attributed to the easier dissociation of Cu(II)/TPZ complex at a lower pH [15]. As a result, GPPCT nanoplatform may decrease the side effects by reducing drug release during body circulation, and release Cu(II) and TPZ quickly at weak acidic tumor sites for efficient CDT and chemotherapy.

Then the generation of ROS by GPPCT in different situations was determined by measuring the degradation of methylene blue (MB) with UV–Vis spectroscopy. As shown in Fig. 2b, the absorption at 664 nm did not change after mixing with only H_2_O_2_ or GPPC for 4 h, indicating the good stability of MB under these conditions. With the addition of H_2_O_2_ and GPPC, the absorption intensity of MB decreased 26.44%. This proved that Cu(II) in GPPC could react with H_2_O_2_ to generate ·OH and induce the degradation of MB. More interestingly, when GSH was added, the degradation rate was accelerated significantly with the increase of GSH concentration. The absorption intensity decreased almost three folds at 10 mM GSH, indicating that about 76.72% of MB had been degraded. This should be mainly due to that GSH may reduce Cu(II) to Cu(I), which has a higher Fenton reaction efficiency. It is worth noting that GPPCT may further enhance the MB degradation to a level of 88.79% with the aid of TPZ, illustrating their capability in massive ·OH production. To verify the function of TPZ, UV–Vis spectra of MB in the presence of TPZ were monitored in Additional file 1: Fig. S8b. It is clearly that TPZ can degrade MB efficiently with/without the existence of H_2_O_2_ and the degradation became more thorough at a high GSH concentration. This result indicated that in the presence of excessive GSH in TME, TPZ may further increase the inhibition effect of GPPCT nanoplatforms at tumor sites. Furthermore, the degradation process of MB over time in Fig. 2c suggested that ·OH groups could be produced continuously by GPPCT nanoplatform in the existence of GSH, implying their long-term and efficient therapeutic effect on malignant tumors. Finally, ROS generation in different pH environments (5.5, 6.5, and 7.4) was evaluated in Fig. 2d. Clearly, the degradation rate of MB enhanced with the decrease of pH, and about 92.12% of MB were degraded at pH 5.5 in the presence of GSH, as a result of considerable ·OH generation by GPPCT in TME. Therefore, the pH responsive drug release property and high toxic radical producing efficiency make GPPCT an excellent nanoplatform for synergistic CDT and chemotherapy.

### Cellular uptake and cytotoxicity of GPPCT

4T1 breast cancer cell lines overexpressing sialic acid residues were chosen as model cells in this study. To simulate hypoxia situation, hypoxia model of 4T1 cells were set by incubating with CoCl_2_·6H_2_O (100 μM) for 24 h according to previous studies [33] (Additional file 1: Fig. S9a), and the expression of hypoxia inducible factor HIF-1α was verified by Western Blot in Additional file 1: Fig. S9b and c. Compared with normoxic 4T1 cells, the HIF-1α expression of hypoxia 4T1 cells enhanced significantly, indicating the successful construction of hypoxia cell model. Therefore, mouse fibroblast L929 cells, normoxic and hypoxic 4T1 cells were applied firstly to assess the biocompatibility of GPP nanocarriers by CCK-8 method. As shown in Additional file 1: Fig. S10, the viability of different cells was higher than 80% after incubation with GPP in the studied concentration range, suggesting the good biocompatibility of GPP as a nanocarrier.

**Fig. 3:**
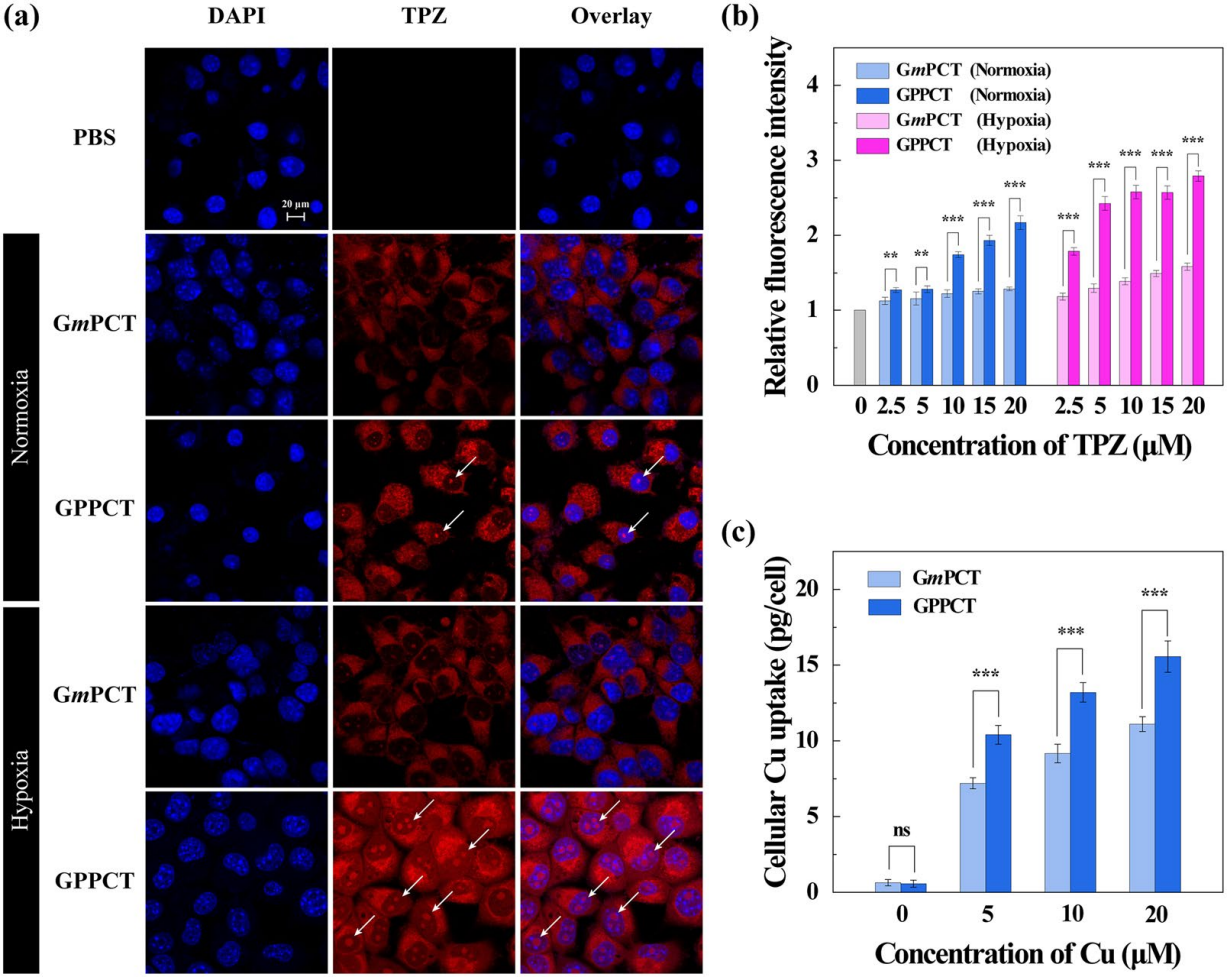
**a** Confocal images of normixic and hypoxic 4T1 cells treated with PBS, GPPCT or G*m*PCT ([TPZ] = 2.5 μM) for 6 h. Cell nucleus was stained with DAPI (Scale bar: 20 μm). **b** Flow cytometry of normoxic and hypoxic 4T1 cells treated with PBS, GPPCT or G*m*PCT at different TPZ concentrations for 6 h. **c** Cellular Cu concentration of 4T1 cells after treated with GPPCT and G*m*PCT at various Cu concentrations for 6 h

To evaluate the targeted delivery and cellular uptake of GPPCT, laser confocal microscope (Fig. 3a) and flow cytometry (Fig. 3b) were used to examine the red fluorescence of TPZ after incubation with normoxic and hypoxic 4T1 cells for 6 h. It could be clearly seen that red fluorescence signals were concentrated well in cytoplasm of both normoxic and hypoxic 4T1 cells after incubation with nanoplatforms, and GPPCT group showed much brighter signals in comparison with G*m*PCT group. This demonstrated that GPPCT could be selectively uptaken by 4T1 cells through the specific recognition between PBA on GPPCT and the overexpressed sialic acid on 4T1 cell membranes. The targeting property of GPPCT was further proved by the result of flow cytometry. As shown in Fig. 3b, the fluorescence intensity of GPPCT group was significantly higher than G*m*PCT group at all TPZ concentrations. Moreover, the fluorescence intensity of GPPCT group in hypoxia 4T1 cells was much stronger than that of normoxic 4T1 cells, and more red spots in the nucleus could be observed in Fig. 3a. These results further proved that TPZ can be activated under hypoxic conditions and enter the nucleus more effectively, which may exert drug activity more efficiently to hypoxic tumors [34]. In addition, the cellular Cu content after incubation with GPPCT and G*m*PCT nanoplatforms was measured by ICP-OES in Fig. 3c in order to further verify their targeting property. With the increase of nanoplatforms concentration, the cellular Cu concentration increased significantly, indicating that both nanoplatforms could be endocytosed into cells due to their suitable nano-size. More importantly, GPPCT nanoplatforms displayed a significantly higher Cu concentration than non-targeted G*m*PCT group (p < 0.001). The selective binding of PBA with sialic acid residues overexpressed on 4T1 cells is expected to be the cause of this difference in cellular uptake. In conclusion, targeted GPPCT nanoplatform can be specifically uptaken by 4T1 cells overexpressing sialic acid residues, and released TPZ could be more efficiently activated in hypoxic tumor cells, which will further enhance the therapeutic effect of GPPCT nanoplatform on hypoxic tumors.

**Fig. 4:**
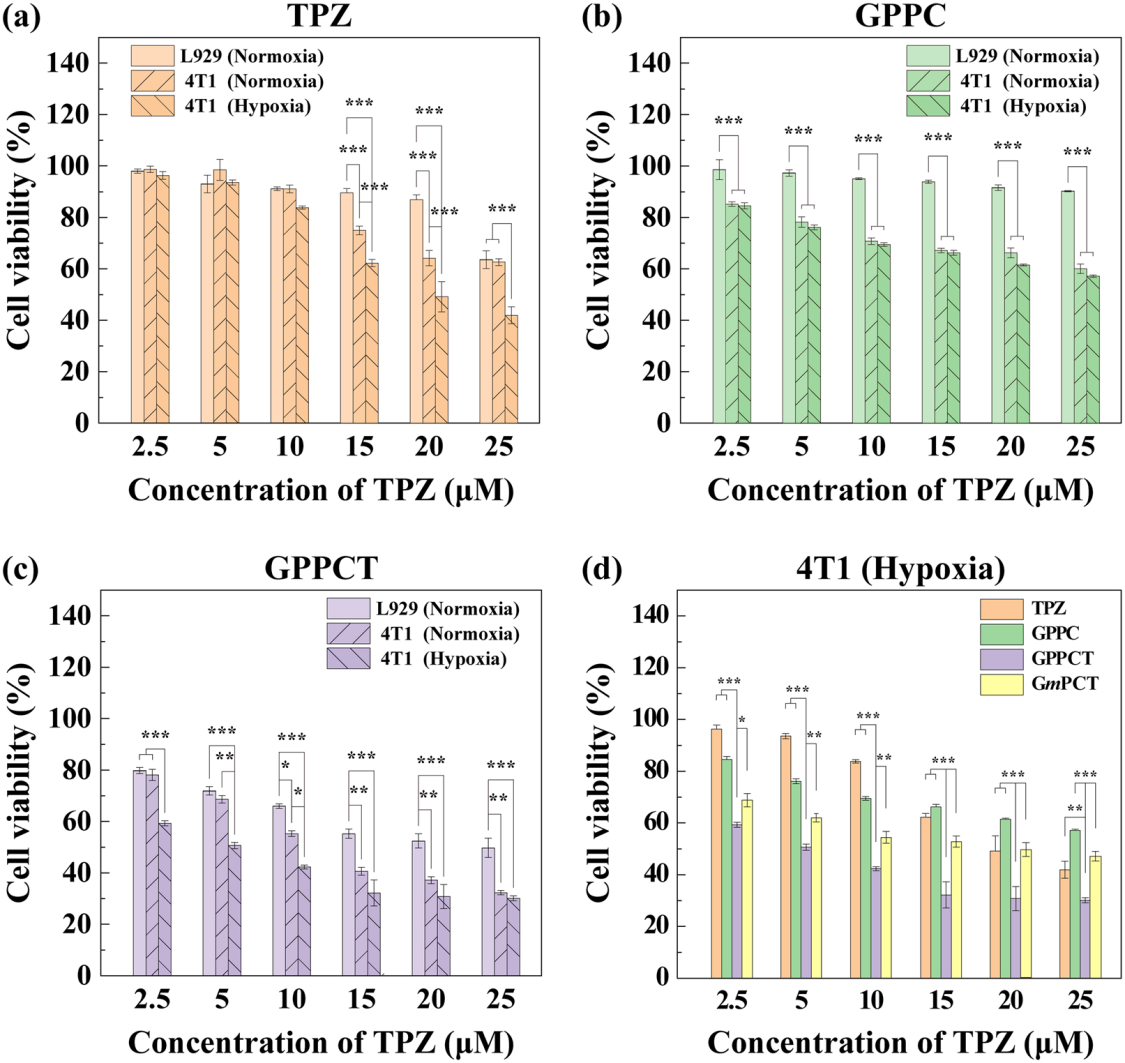
Cell viability of L929 (normoxia) cells and 4T1 (hypoxia and normoxia) cells after incubation with **a** TPZ, **b** GPPC and **c** GPPCT for 24 h detected by CCK-8 assay. **d** Cell viability of hypoxic 4T1 cells after incubation with free TPZ, GPPC, GPPCT and G*m*PCT for 24 h

Subsequently, the viabilities of L929, normoxic and hypoxic 4T1cells were evaluated by incubating with TPZ, GPPC, GPPCT and G*m*PCT at different TPZ concentrations (0, 2.5, 5, 10, 15, 20, 25 μM) for 24 h (Fig. 4 and Additional file 1: Fig. S11). For free TPZ (Fig. 4a), the viability of all three kinds of cells decreased with the increase of drug concentration, and hypoxic 4T1 cells displayed a much lower viability than normoxic 4T1 cells and L929 cells at TPZ concentration over 10 μM. This difference should be attributed to the responsive formation of highly toxic TPZ radicals within hypoxic cells, which will in turn induce single-strand/double-strand DNA breakage through the mediation of topoisomerase II and chromosomal destruction [35–37]. In Fig. 4b, GPPC nanoplatforms displayed obvious toxicity to both normoxic and hypoxic 4T1 cells, but almost no inhibition to mouse fibroblast L929 cells was observed. This result suggested that Cu(II) complexed in GPPC could exert inhibitory effect specifically on tumor cells with high endogenous H_2_O_2_ level, and do less harm to normal cells. In Fig. 4c, the inhibitory effect of GPPCT between different cell lines were systemically compared. L929 cells displayed the highest cell viability, while the viability of both hypoxic and normoxic 4T1 cells decreased rapidly with the increase of drug concentration, indicating that GPPCT nanoplatforms could display the most prominent inhibition effect specific to tumor cells. Hypoxic 4T1 cells displayed a significantly poorer viability than normoxic 4T1 cells at a low GPPCT concentration, possibly due to that hypoxic-sensitive TPZ could be converted to toxic radicals responsively within hypoxic cells and enhance the therapeutic effect. Figure 4d evaluated the inhibition effect of TPZ, GPPC, G*m*PCT, and GPPCT on hypoxic 4T1 cells. It is clearly that GPPCT displayed a significantly lower viability than TPZ or GPPC group alone at the studied concentrations (p < 0.001), verifying their synergistic effect of hypoxia-enhanced chemotherapy and CDT on hypoxic tumor cells. In addition, the cell viability of GPPCT group was significantly lower than G*m*PCT group, suggesting that GPPCT nanoplatform could target tumor cells overexpressing sialic acid residues and efficiently induce cell apoptosis. In contrast, the inhibition effect of GPPCT and G*m*PCT on normoxic 4T1 cells did not display significant difference at a low drug concentration (Additional file 1: Fig. S11) due to the poor activity and toxicity of TPZ at normoxic situation. The IC_50_ values of GPPCT to different cells were calculated in Additional file 1: Table S3. The IC_50_ value of GPPCT on L929 cells was as high as 24.05 μM, which was 2.1 times higher than normoxic 4T1 cells (11.70 μM), and 4.8 times higher than hypoxic 4T1 cells (5.03 μM). This result further demonstrated that GPPCT could specifically inhibit tumor cells, and exhibit an enhanced therapeutic effect on hypoxic cells which are less susceptible to general chemotherapy. The safety indexes of hypoxic and normoxic 4T1 cells were 4.78 and 2.06, respectively, verifying again the good safety of the nanoplatforms. In conclusion, GPPCT can be used as an ideal targeted nanoplatform for the synergistic hypoxia-responsive chemotherapy and CDT of cancer cells overexpressing sialic acid residues.

### Determination of ROS and GSH/GSSG levels in cells

**Fig. 5:**
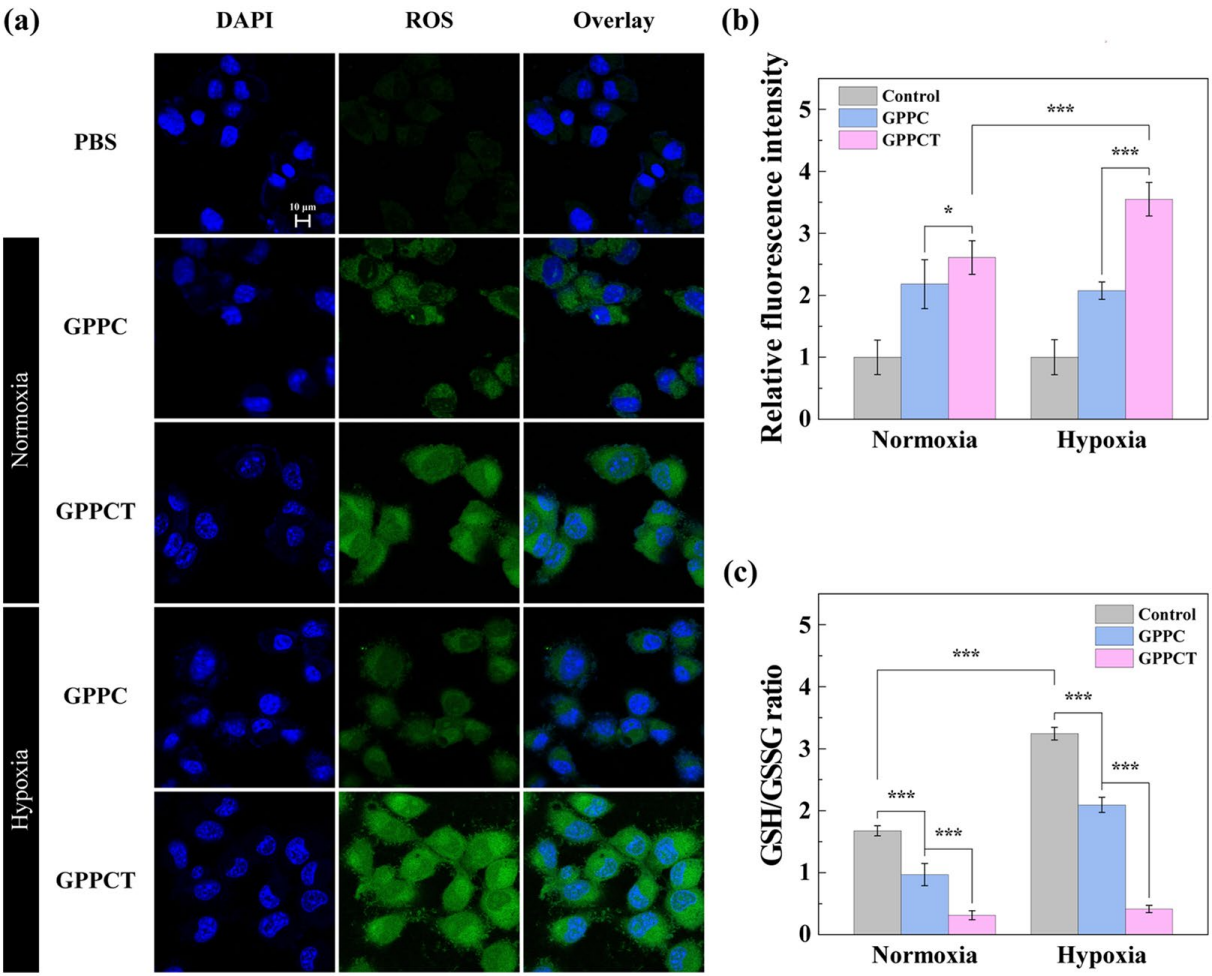
**a** Confocal images of normixic and hypoxic 4T1 cells treated with PBS, GPPC or GPPCT for 6 h. Cell nucleus was stained with DAPI (Scale bar: 10 μm). **b** Intracellular ROS generation and **c** GSH/GSSG ratio in normoxic and hypoxic 4T1 cells treated with PBS, GPPC or GPPCT for 6 h. For **a**–**c**, [TPZ] = 2.50 μM, equivalent to [Cu(II)] = 7.69 μM

In order to better illustrate the antitumor mechanism of GPPCT nanoplatforms at cellular level, 2′,7′-dichlorofluorescin diacetate (DCFH-DA) was used as a fluorescent indicator to detect reactive oxide species formed in cells [38, 39]. In Fig. 5a, 4T1 cells incubated with GPPC showed obvious green fluorescence signals in comparison with PBS control. This result indicated that toxic ·OH were generated by Fenton-like reaction between Cu(II) released from GPPC nanoplatform and endogenous H_2_O_2_, inducing the formation of 2′,7′-dichlorofluorescein (DCF) with fluorescence in cells. The green fluorescence in cells treated with GPPCT under normoxic or hypoxic conditions was much brighter than that of GPPC group, suggesting that TPZ can produce additional reactive radicals, in consistent with the result in Fig. 2b and Additional file 1: Fig. S8b. And the hypoxic 4T1 cells showed much stronger fluorescence signal than normoxic 4T1 cells due to the easier activation of TPZ under hypoxia condition, which would lead to the formation of toxic BTZ radical and ·OH. Figure 5b showed the quantitative analyzation of fluorescence intensity by flow cytometry. After treated with nanoplatform, 4T1 cells displayed an enhanced fluorescence intensity, and GPPCT caused a much stronger fluorescence signal than GPPC in both hypoxic (p < 0.001) and normoxic 4T1 cells (p < 0.05) due to the encapsulation of TPZ. More importantly, the fluorescence intensity in hypoxic 4T1 cells of GPPCT group was much higher than that in normoxic 4T1 cells, while no significant difference was observed in GPPC treated 4T1 cells. This further demonstrated that TPZ can produce more reactive radicals under hypoxia conditions, which may boost the therapeutic effect of GPPCT on hypoxia tumors. Moreover, the GSH content in 4T1 cells under normoxic and hypoxic conditions was measured to further verify the function of GSH in tumor treatment. As shown in Fig. 5c, GSH content in hypoxic 4T1 cells was significantly higher than normoxic ones, which would facilitate the reduction of Cu(II) to Cu(I) and promote the Fenton reaction efficiency for CDT. After incubation with GPPC, the GSH content in normoxic and hypoxic cells decreased significantly (p < 0.001) due to the consumption of GSH for Cu(II)/Cu(I) reduction. In comparison with GPPC, the GSH/GSSH ratio of normoxic and hypoxic cells decreased significantly after GPPCT treatment (p < 0.001), suggesting that GSH played an important role in the activation of TPZ in hypoxic cells. As a result, the excessive GSH in tumor cells could be depleted by the reduction of Cu(II) to Cu(I) and converting TPZ to active radicals, which may in turn break the redox homeostasis in tumor cells, and improve the CDT efficacy. In conclusion, the GPPCT nanoplatform may efficiently produce toxic ROS, reduce the GSH level in cancer cells, and successfully enhance the therapeutic effect to tumors.

### In vivo antitumor effect of GPPCT

**Fig. 6:**
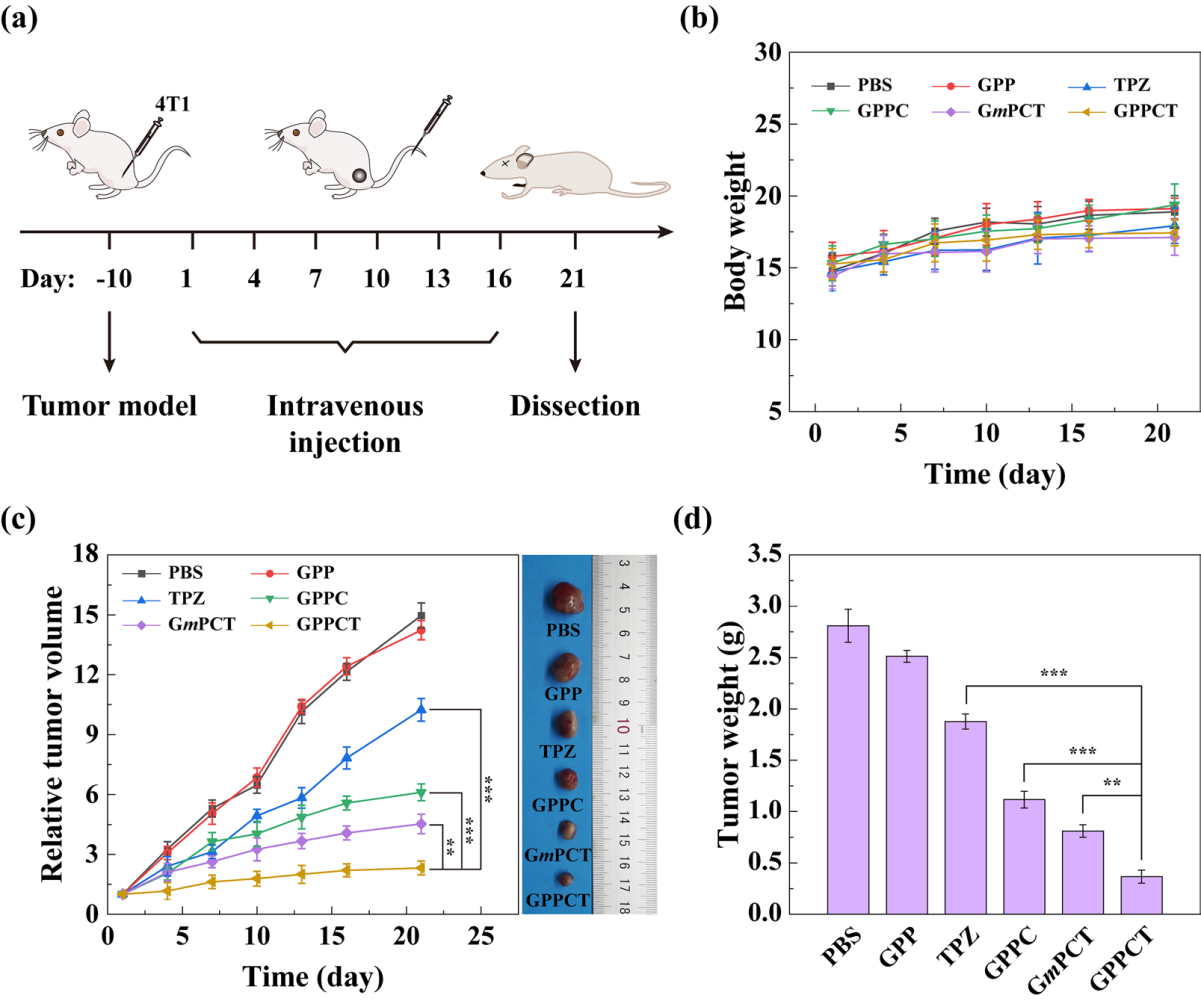
**a** Treatment schedule of the in vivo antitumor experiment ([TPZ] = 2 mg/kg, 100 μL). **b** Body weight, **c** relative tumor volume, representative tumor photographs and **d** tumor weight of mice after different treatments (n = 5)

To verify the therapeutic effect of GPPCT in vivo, BALB/c nude mice transplanted with 4T1 tumors were randomly divided into 6 groups (PBS, GPP, GPPC, CPPCT, G*m*PCT, and TPZ, n = 5) when the tumor size reached about 200 mm^3^. Intravenous administration was planned on day 1, 4, 7, 10, 13, and 16 (Fig. 6a), and body weight (Fig. 6b) and relative tumor volume (Fig. 6c and Additional file 1: Fig. S12) were measured every 3 days. There were no mice dead in all groups during the treatment, and the body weight showed no significant decrease as depicted in Fig. 6b, indicating that nanoplatforms had no obvious toxic and side effect. As shown in Fig. 6c, the relative tumor volume of PBS and GPP groups increased by 14–15 times, indicating that pure nanocarrier GPP had no inhibition effect on tumors. Although the relative tumor size of free TPZ group displayed a lower tumor growth rate as 10.24 times, the therapeutic effect is still quite poor possibly due to its fast metabolism in blood circulation and the low uptake in tumor cells. In contrast, GPPC group showed a significantly better inhibition effect with a tumor volume increase of only 6.27 times (p < 0.001). This should be attributed to that targeted GPPC nanoplatforms could effectively generate toxic ·OH at tumor sites for CDT. More encouragingly, the relative tumor size of GPPCT group was only about 51% of G*m*PCT group, 22% of TPZ group and 37% of GPPC group (p < 0.001). The magnificent inhibition effect of GPPCT could be mainly attributed to the following two factors: (1) the specific accumulation and efficient uptake of drugs at tumors, and (2) the synergistic therapeutic effect of CDT by Cu(II) and hypoxia-enhanced chemotherapy by TPZ. After all mice were sacrificed on day 21, tumor tissues were extracted and weighted as shown in Fig. 6d. Among different groups, the tumor weight of GPPCT group was the lowest. All these results clearly demonstrated the outstanding in vivo antitumor effect of targeted GPPCT nanoplatforms could effectively inhibit the growth of sialic acid overexpressed 4T1 tumors as a result of the synergistic therapeutic effect of hypoxia-enhanced chemotherapy and CDT.

**Fig. 7:**
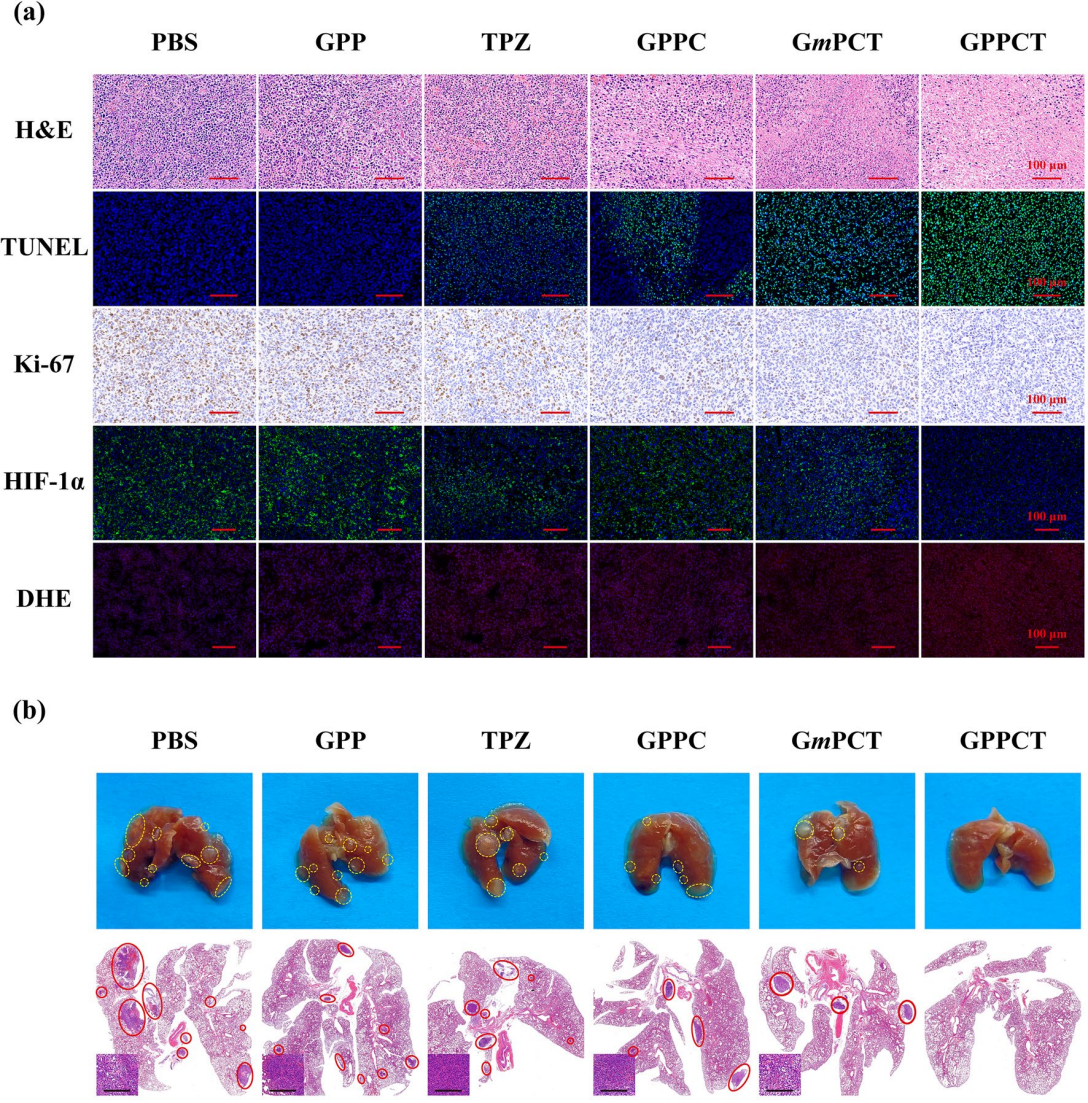
**a** Images of tumor sections of different treatments after H&E staining, immunofluorescence staining of TUNEL (green), Ki-67 staining, fluorescence staining of HIF-1α (green) and DHE (red). **b** Representative lung photographs and H&E-stained lung sections of different treatments (Inset: magnified sections, Scale bar bar:100 μm)

To further illustrate the therapeutic mechanism in vivo, H&E, TUNEL, Ki-67, HIF-1α, and DHE staining were performed on tumor sections as shown in Fig. 7a. In H&E staining image, the reduced blue signal indicated that most tumor tissues in GPPCT group were necrotic. In the TUNEL staining image, an enhancing green fluorescence signal was observed from TPZ, GPPC, G*m*PCT to GPPCT group, suggesting that more DNA fragments were formed by GPPCT treatment. According to Additional file 1: Fig. S13a, the apoptosis rate of GPPCT group was calculated to be 95.90%, which was significantly higher than that of G*m*PCT (78.48%), GPPC (48.92%), and free TPZ (30.91%) group (p < 0.001). In the Ki-67 antibody staining assay, a similar ascending sequential order of cell proliferation was observed, and GPPCT displayed the lowest Ki-67 expression level as 8.37% (Additional file 1: Fig. S13b). Since TPZ could be metabolized in hypoxic cells to produce DNA-damaging radicals and kill hypoxic cells selectively, the expression of HIF-1α in tumors was detected by hypoxyprobe immunofluorescence assay to assess the ratio of hypoxic cells in tumor region in Fig. 7a. Strong green fluorescence signal of HIF-1α could be observed in the tumor section of PBS group, which indicated that large number of hypoxic cells existed in solid tumor in vivo by the rapid growth of tumor. In contrast, the tumor section after GPPCT treatment displayed almost no green fluorescence signal. As calculated in Additional file 1: Fig. S13c, the HIF-1α expression level of GPPCT group was only 5.52% of PBS group, while that of TPZ, GPPC, and G*m*PCT groups was 37.90%, 49.25%, and 31.19%, respectively. This result again demonstrated that GPPCT could effectively inhibit hypoxic tumors in vivo by specific targeting and combination therapy. In addition, local ROS production in tumor region was also detected by using dihydroethidium (DHE) as a fluorescence probe in Fig. 7a. For GPPCT, G*m*PCT, GPPC, and free TPZ groups, the red fluorescence signals appeared in tumor section resulting from the generation of ROS at tumor sites in vivo. The average red fluorescence intensity of GPPCT group was significantly higher than that of free TPZ, GPPC, and G*m*PCT groups (p < 0.001) as shown in Additional file 1: Fig. S13d. This result verified that GPPCT nanoplatforms could produce toxic ROS more efficiently at tumor than other groups, which should be mainly due to three reasons. Firstly, GPPCT could specifically accumulate at tumor sites and be efficiently uptaken by tumor cells via the binding between PBA on GPPCT and sialic acid residues overexpressed on 4T1 cells for the targeted delivery of Cu(II) and TPZ. Secondly, the excessive intracellular GSH would be depleted by reducing Cu (II) to Cu(I) and activating TPZ in hypoxic tumor cells, which suppresses the cellular antioxidative system for ROS scavenging. Thirdly, abundant ·OH radicals would be produced via the Fenton-like reaction between Cu(I) and endogenous H_2_O_2_, and effectively inhibit solid tumor with the hypoxia-activated TPZ radicals in a synergistic manner.

Considering the highly invasive and aggressive nature of breast cancer 4T1 model, the lung tissues in different groups were collected and H&E stained to assess tumor metastasis to distant organs (Fig. 7b). A large number of metastatic sites could be clearly seen in the lungs of PBS and GPP group, while the treatment groups of TPZ and GPPC displayed a much lower number of pulmonary metastatic sites. Notably, compared with G*m*PCT group, GPPCT group showed a relatively intact lung structure with almost no metastasis, demonstrating the tumor invasion could be effectively inhibited by GPPCT. Therefore, GPPCT nanoplatforms could be an effective antitumor strategy to inhibit the growth and metastasis of tumors by the combination of hypoxia-enhanced chemotherapy and CDT.

### In vivo biodistribution and biosafety of GPPCT

**Fig. 8:**
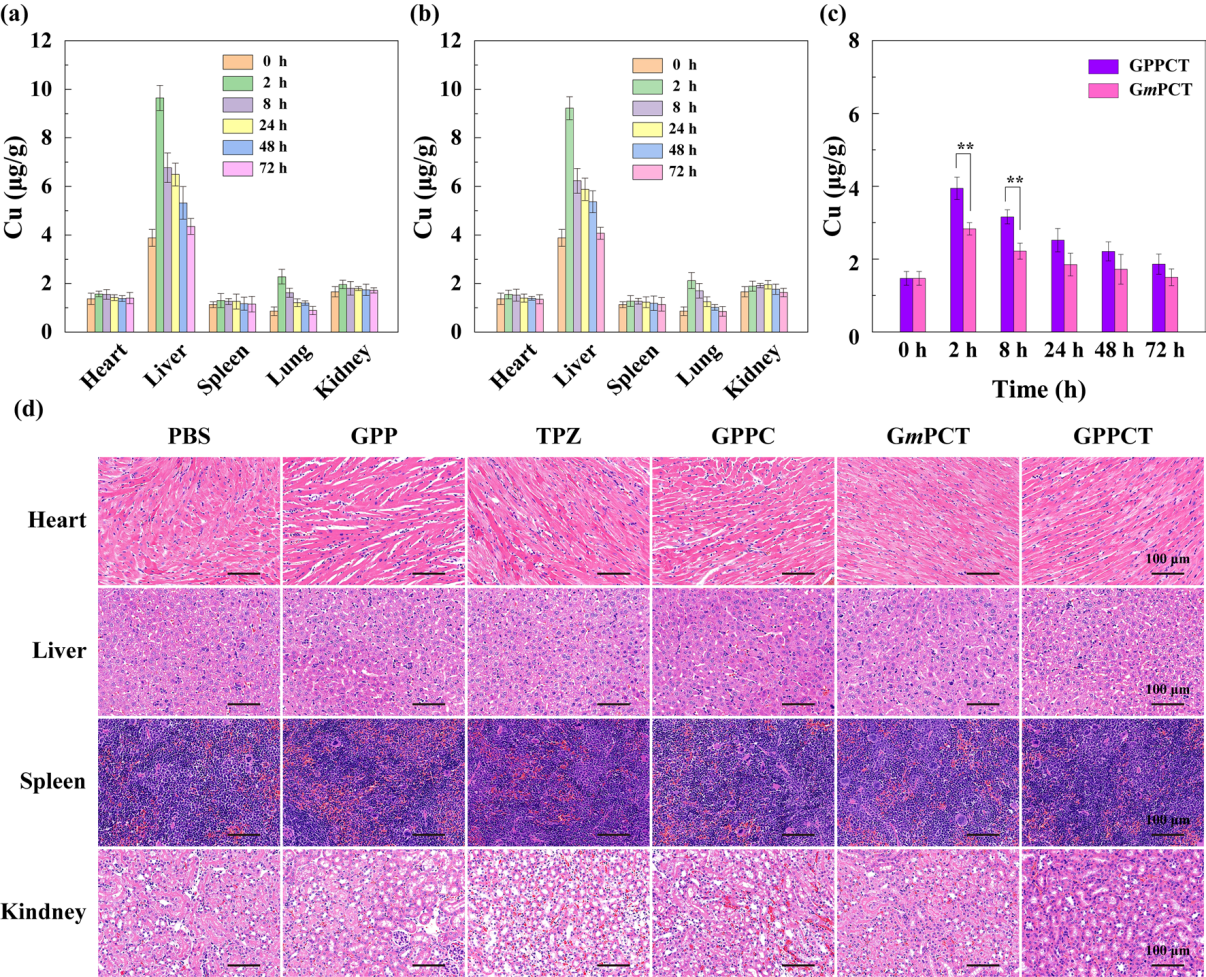
Biodistribution of Cu in major organs of mice post-intravenous injection of **a** GPPCT and **b** G*m*PCT at different time points. **c** Cu concentration in tumors post-intravenous injection of GPPCT and G*m*PCT. For **a**–**c**, [Cu(II)] = 6.9 mM, equivalent to [TPZ] = 2 mg/kg for each mouse. **d** H&E-stained slices of major organs of 4T1 tumor-bearing mice after different treatments

To understand the biological distribution of GPPCT and G*m*PCT in vivo, the contents of copper in heart, liver, spleen, lung, kidney and tumors at different time intervals were determined by ICP-OES (Fig. 8a–c). For both targeted and non-targeted nanoplatforms, the Cu content in liver, lung and tumor increased significantly after intravenous injection, and peaked at 2 h. Then the Cu concentration in all major organs decreased to normal level after 72 h by metabolization, implying that the injected nanoplatforms were almost completely cleared from the body within 3 days. It is worth noting that the Cu content in tumor tissue of targeted GPPCT group was significantly higher than G*m*PCT group within 8 h after injection (p < 0.01). This result indicated that GPPCT nanoplatforms could more effectively accumulate at tumor sites by the specific recognition of PBA to sialic acid resides overexpressed on 4T1 cancer cells, and be safely excreted from the body.

The biotoxicity of nanoplatforms to major organs after different treatments was evaluated by H&E staining assays shown in Fig. 8d. No apparent inflammation or cell necrosis and apoptosis were observed in heart, liver, spleen, and kidney, indicating the good biocompatibility of GPPCT nanoplatforms. Finally, the routine blood and blood biochemical analysis were performed after GPPCT treatment [38]. No obvious abnormality was observed in Additional file 1: Fig. S14, suggesting the good compatibility of GPPCT, and the proper liver and kidney function after GPPCT treatment. In conclusion, the biodistribution study of GPPCT nanoplatforms further confirmed the targeting effect to tumor overexpressing sialic acid residues, and they can be metabolized without significant damage to normal organs, which is of great importance for in *vivo* biomedical applications.

## Conclusions

In summary, a TME-responsive and targeted GPPCT nanoplatform was synthesized for the combination of hypoxia-sensitive chemotherapy and CDT for tumors overexpressing sialic acid residues. The GPPCT nanoplatform has good colloidal stability, high drug loading capacity and efficiency, and pH-sensitive drug release property. In vitro experiments demonstrated that GPPCT could be specifically uptaken by cancer cells overexpressing sialic acid residues, effectively generate ROS within cells and deplete GSH, and significantly kill hypoxic tumor cells because of the activation of TPZ under a hypoxic situation. In vivo results showed that GPPCT nanoplatforms could be specifically accumulated at tumor sites after long circulation, and effectively inhibit the growth and metastasis of 4T1 breast tumor by the combination of CDT and chemotherapy. Moreover, they can be metabolized with no significant systemic toxicity. Therefore, in this study, the targeted GPPCT nanoplatform with synergistic CDT and hypoxia-responsive chemotherapy provides an efficient and safe strategy for therapy of tumors and tumor metastasis.

## Materials and methods

Please see Additional file 1.

## Supplementary Information

**Additional file 1: Table S1.** The TPZ drug loading content (LC %) and entrapment efficiency (EE %) of GPPCT and G*m*PCT. **Table S2.** The hydrodynamic diameters, polydispersity index and Zeta potential of GPP, G*m*P, GPPC, G*m*PC, GPPCT and G*m*PCT. **Table S3.** IC_50_ value and safety index of GPPCT for different cells. **Fig. S1.** The action mechanism of TPZ under normal and hypoxic conditions. **Fig. S2.** ^1^H NMR spectra of (a) PBA-PEG-COOH, (b) G5.NH_2_-PEG-PBA, (c) G5.NH_2_-*m*PEG and (d) G5.NHAc-*m*PEG. **Fig. S3.** Schematic diagram of the synthesis of G5.NHAc-*m*PEG@Cu(II)/TPZ (G*m*PCT). **Fig. S4.** The UV–vis spectra of G*m*P, G*m*PC, G*m*PCT, CuCl_2_ and TPZ. **Fig. S5.** (a) TEM image and (b) size distribution histograms of G*m*PCT. **Fig. S6.** Hydrodynamic diameter histogram of GPPCT and G*m*PCT. **Fig. S7.** Changes of hydrodynamic diameters and Zeta potential of (a, b) GPPCT and (c, d) G*m*PCT in water, PBS and DMEM medium for 1, 3, 5 and 7 days. **Fig. S8.** (a) Accumulative release of TPZ from GPPCT in buffer solutions with different pH of 5.5, 6.5 and 7.4 at 37 ℃ ([GPPCT] = 1 mg/mL). (b) UV–vis spectra of MB treated with H_2_O_2_ and TPZ with or without the presence of GSH for 4 h ([TPZ] = 200 μM). **Fig. S9.** (a) CCK-8 assay of 4T1 cells under different concentrations of CoCl_2_·H_2_O in the presence or absence of GPPCT ([TPZ] = 2.5 μM). (b) HIF-1α expression of 4T1 cells in the presence or absence of CoCl_2_·H_2_O determined by western blot and (c) its quantitative analysis results ([CoCl_2_·H_2_O] = 100 μM). **Fig. S10.** Cell viability of 4T1 (hypoxia and normoxia) and L929 (normoxia) cells after co-culture with GPP detected by CCK-8 assay. **Fig. S11.** *m*Cell viability of (a) L929 cells and (b) normoxic 4T1 cells after co-culture with free TPZ, GPPC, GPPCT and GmPCT for 24 h detected by CCK-8 assay. **Fig. S12.** Representative images of tumor bearing mice with different treatments on the 20th days post-treatment. (1: PBS, 2: GPP, 3: TPZ, 4: GPPC, 5: G*m*PCT and 6: GPPCT.). **Fig. S13.** Quantification of (a) TUNEL mean fluorescence intensity, (b) positive signals of Ki-67, (c) HIF-1α and (d) DHE mean fluorescence intensity after different treatments. *** p < 0.001. **Fig. S14.** Blood biochemistry analysis of (a) WBC, (b) RBC, (c) HGB, (d) MCH, (e) LYM, (f) HCT, (g) MCV, (h) MCHC, (i) PTL, (j) MON and (k) GRAN of mice on the last day of different treatments of (I) PBS and (II) GPPCT. Liver function index of (l) ALT and (m) AST and kidney function index of (n) UREA, (o) UA and (p) CREA of mice on the last day of with different treatments of (I) PBS and (II) GPPCT (n = 3).

## Data Availability

All data analyzed during this study are included in this published article and its supplementary information files.

## Abbreviations

TME: Tumor microenvironment
H_2_O_2_: Hydrogen peroxide
GSH: Glutathione
CDT: Chemodynamic therapy
ROS: Reactive oxygen species
·OH: Hydroxyl radicals
pH: Potential of hydrogen
NPs: Nanoparticles
TPZ: Tirapazamine
BTZ: Benzotriazinyl
PEG: Polyethylene glycol
PBA: Phenylboronic acid
PAMAM: Poly(amidoamine)
GPPCT: G5.NHAc-PEG-PBA@Cu(II)/TPZ
G*m*PCT: G5.NHAc-*m*PEG@Cu(II)/TPZ
4-CPBA: 4-Hydroxyphenylboronic acid
GPP: G5.NHAc-PEG-PBA
G*m*P: G5.NHAc-*m*PEG
LC: Loading content
GPPC: G5.NHAc-PEG-PBA@Cu(II)
G*m*PC: G5.NHAc-*m*PEG@Cu(II)
EE: Entrapment efficiency
ICP-OES: Inductively coupled plasma-optical emission spectroscopy
MB: Methylene blue
CCK-8: Cell counting kit-8
DCFH-DA: 2’,7’-Dichlorofluorescin diacetate
DCF: 2’,7’-Dichlorofluorescein
PBS: Phosphate buffer saline
H&E: Hematoxylin–eosin staining
TUNEL: TdT-mediated dUTP Nick-End Labeling
HIF-1α: Hypoxia-inducible factor-1α
DHE: Dihydroethidium
EDC: 1-Ethyl-3-(3-dimethylaminopropyl)carbodiimide
NHS: N-Hydroxysuccinimide
MWCO: Molecular weight cut-off
DMSO: Dimethyl sulfoxide
EDTA: Ethylenediaminetetraacetic acid
DMEM: Dulbecco’s modified eagle medium
DAPI: 4,6-Diamino-2-phenyl indole
^1^H NMR: Proton nuclear magnetic resonance
UV–Vis: Ultraviolet–visible spectroscopy
TEM: Transmission electron microscopy
DLS: Dynamic light scattering
WBC: White blood cells
RBC: Red blood cells
HGB: Hemoglobin
MCH: Means corpuscular hemoglobin
LYN: Lymphocytes percentage
HCT: Hematocrit
MCV: Means corpuscularvolume
MCHC: Means corpuscular hemoglobin concentration
PLT: Platelets
MON: Monocyte
GRAN: Granulocyte
ALT: Alanine aminotransferase
AST: Aspartate aminotransferase
UREA: Urea
UA: Uric acid
CREA: Creatinine
