# Supplementary material for: A tumor microenvironment-responsive poly(amidoamine) dendrimer nanoplatform for hypoxia-responsive chemo/chemodynamic therapy
Source: J Nanobiotechnology. 2022 Jan 21;20:43. doi: 10.1186/s12951-022-01247-6 (PMC8781438; doi:10.1186/s12951-022-01247-6)
Supplement: Supplementary file 1 — Additional file 1: Table S1. The TPZ drug loading content (LC %) and entrapment efficiency (EE %) of GPPCT and GmPCT. Table S2. The hydrodynamic diameters, polydispersity index and Zeta potential of GPP, GmP, GPPC, GmPC, GPPCT and GmPCT. Table S3. IC50 value and safety index of GPPCT for different cells. Fig. S1. The action mechanism of TPZ under normal and hypoxic conditions. Fig. S2. 1H NMR spectra of (a) PBA-PEG-COOH, (b) G5.NH2-PEG-PBA, (c) G5.NH2-mPEG and (d) G5.NHAc-mPEG. Fig. S3. Schematic diagram of the synthesis of G5.NHAc-mPEG@Cu(II)/TPZ (GmPCT). Fig. S4. The UV–vis spectra of GmP, GmPC, GmPCT, CuCl2 and TPZ. Fig. S5. (a) TEM image and (b) size distribution histograms of GmPCT. Fig. S6. Hydrodynamic diameter histogram of GPPCT and GmPCT. Fig. S7. Changes of hydrodynamic diameters and Zeta potential of (a, b) GPPCT and (c, d) GmPCT in water, PBS and DMEM medium for 1, 3, 5 and 7 days. Fig. S8. (a) Accumulative release of TPZ from GPPCT in buffer solutions with different pH of 5.5, 6.5 and 7.4 at 37 ℃ ([GPPCT] = 1 mg/mL). (b) UV–vis spectra of MB treated with H2O2 and TPZ with or without the presence of GSH for 4 h ([TPZ] = 200 μM). Fig. S9. (a) CCK-8 assay of 4T1 cells under different concentrations of CoCl2·H2O in the presence or absence of GPPCT ([TPZ] = 2.5 μM). (b) HIF-1α expression of 4T1 cells in the presence or absence of CoCl2·H2O determined by western blot and (c) its quantitative analysis results ([CoCl2·H2O] = 100 μM). Fig. S10. Cell viability of 4T1 (hypoxia and normoxia) and L929 (normoxia) cells after co-culture with GPP detected by CCK-8 assay. Fig. S11. mCell viability of (a) L929 cells and (b) normoxic 4T1 cells after co-culture with free TPZ, GPPC, GPPCT and GmPCT for 24 h detected by CCK-8 assay. Fig. S12. Representative images of tumor bearing mice with different treatments on the 20th days post-treatment. (1: PBS, 2: GPP, 3: TPZ, 4: GPPC, 5: GmPCT and 6: GPPCT.). Fig. S13. Quantification of (a) TUNEL mean fluorescence intensity, (b) pos [file 12951_2022_1247_MOESM1_ESM.pdf]

Additional file

For

A Tumor Microenvironment-Responsive Poly(amidoamine) Dendrimer Nanoplatform for Hypoxia-Responsive Chemo/Chemodynamic Therapy

Experimental section

Materials

4-Carboxyphenylboronicacid and polyethylene glycol (NH_2-PEG-COOH 2000 and *m*PEG-COOH 2000) were purchased from Yarebio (Shanghai, China). 1-Ethyl-3-(3-dimethylaminopropyl) carbodiimide (EDC), N-Hydroxysuccinimide (NHS) and Copper(II) chloride dihydrate were obtained from J&K Chemical (Beijing, China). Generation 5 poly(amidoamine) (G5 PAMAM) dendrimer was bought from Dendritech (Michigan, America). Laboratory reagent dimethyl sulfoxide (DMSO) was obtained from Titan (Shanghai, China). Triethylamine and acetic anhydride were purchased from Sinopharm Chemical Reagent Co., Ltd (Shanghai, China). Tirapazamine (TPZ) was obtained from Shanghai Macklin Biochemical Co., Ltd (Shanghai, China). Cobalt(II)chloride hexahydrate ($CoCl_2 \cdot 6H_2O$) was purchased from Sigma-Aldrich (Missouri, America). DMEM high glucose culture medium, 0.25 % trypsin & 0.02 % ethylenediaminetetraacetic acid (EDTA), fetal bovine serum and penicillin-streptomycin solution were purchased from Genom (Hangzhou, China). Regenerated cellulose dialysis bags with a molecular weight cut-off (MWCO) at 8000~14000 and 500 were acquired from Shanghai Yuanye Biotechnology Corporation (Shanghai, China). Cell Counting Kit-8, GSH and GSSG Assay Kit, Reactive Oxygen Species Assay Kit were purchased from Beyotime Biotechnology (Shanghai, China). DAPI staining kit was purchased from BestBio (Shanghai, China). Water used in all experiment was purified using a PURIST UV Ultrapure Water System (RephiLe Bioscience, Ltd., Shanghai, China) with a resistivity higher than 18.2 MΩ·cm. All chemical reagents are commercially available and can be used without further purification, unless otherwise stated.

Synthesis of G5.NHAc-PEG-PBA (GPP)

PBA was modified onto the surface of G5 PAMAM through PEG to construct the targeted dendrimer nanocarriers (GPP). First, EDC (191.7 mg) and NHS (115.1 mg) was added into PBA (33.2 mg) under

stirring at room temperature for 4 h. Then, the activated PBA was slowly added into NH_2-PEG (40 mg) and stirred for 3 days. At the end of the reaction, the reaction solution was transferred to a dialysis bag with a MWCO of 500 for dialysis in ultrapure water for 2 days. Finally, the solid product PBA-PEG-COOH was obtained by lyophilization. The PBA-PEG-COOH (27.1 mg) obtained above was activated by EDC (26 mg)/NHS (15.6 mg), and then the activated PBA-PEG-COOH was added to G5 PAMAM (34.2 mg) and stirred for 3 days. After the reaction, a dialysis bag with a MWCO of 8000~14000 was used for dialysis in ultra-pure water for 3 days. Finally, the solid product G5.NH_2-PEG-PBA was obtained by lyophilization. The obtained G5.NH_2-PEG-PBA was then acetylated according to previous reports [1]. Add triethylamine (147 μL) slowly to G5.NH_2-PEG-PBA (60 mg) under stirring at room temperature for 30 min, then add acetic anhydride (83 μL) to the above solution and stirred at room temperature for 24 h. The solid G5.NHAc-PEG-PBA (GPP) was obtained by dialysis, lyophilization according to the above methods, which was stored at -20 °C.

Synthesis of G5.NHAc-PEG-PBA@Cu(II) (GPPC)

Cu(II) is loaded into GPP by the complexation of amino group and copper ion. The $CuCl_2$ solution (3.9 mg, 100 μL water) was added to the GPP solution (40 mg) in ultrasound and mixed evenly. The reaction solution was centrifuged at 7000 g for 15 min in an ultrafiltration centrifuge tube with a MWCO of 10000. Then the product was re-dispersed in 4 mL ultrapure water to obtain G5.NHAc-PEG-PBA@Cu(II) (GPPC) solution with a concentration of about 10 mg/mL, and stored at 4 °C.

Preparation of G5.NHAc-PEG-PBA@Cu(II)/TPZ (GPPCT)

Cu(II)/TPZ complex is formed through the coordination of copper ions with TPZ and is wrapped in GPP. The TPZ solution (1.5 mg) was added to the GPPC solution (3.5 mL), and the reaction was carried out overnight by magnetic stirring at room temperature. Ultrafiltration centrifugation as described above, and the products were re-dispersed in 3.5 mL ultrapure water to obtain G5.NHAc-

PEG-PBA@Cu(II)/TPZ (GPPCT) solution with a concentration of 10 mg/mL.

Synthesis of G5.NHAc-*m*PEG (G*m*P)

The G*m*P was synthesized according to the stepwise procedures similarly to the synthesis of G5.NHAc-PEG-PBA (GPP). G5.NH_2-*m*PEG was first prepared according to previous study [2], in which *m*PEG-COOH (27 mg) was activated by EDC (25.9 mg)/NHS (15.5 mg), and then the activated *m*PEG-COOH was added to G5 PAMAM (35.1 mg) and stirred for 3 days. After the reaction, a dialysis bag with a MWCO of 8000~14000 was used for dialysis in ultra-pure water for 3 days. Add triethylamine (145.5 μL) slowly to G5.NH_2-*m*PEG (57.5 mg) under stirring at room temperature for 30 min, then add acetic anhydride (82.4 μL) to the above solution and stirred at room temperature for 24 h. The solid G5.NHAc-*m*PEG (G*m*P) was obtained by dialysis, lyophilization according to the above methods, which was stored at -20 °C.

Synthesis of G5.NHAc-*m*PEG@Cu(II) (G*m*PC)

The $CuCl_2$ solution (3.5 mg, 100 μL water) was added to the G*m*P solution (35 mg) in ultrasound and mixed evenly. The reaction solution was centrifuged at 7000 g for 15 min in an ultrafiltration centrifuge tube with a MWCO of 10000. Then the product was re-dispersed in 3.75 mL ultrapure water to obtain G5.NHAc-*m*PEG@Cu(II) (G*m*PC) solution with a concentration of about 10 mg/mL, and stored at 4 °C.

Synthesis of G5.NHAc-*m*PEG@Cu(II)/TPZ (G*m*PCT)

The TPZ solution (1.34 mg) was added to the GPPC solution (3 mL), and the reaction was carried out overnight by magnetic stirring at room temperature. Ultrafiltration centrifugation as described above, and the products were re-dispersed in 3 mL ultrapure water to obtain G5.NHAc-*m*PEG@Cu(II)/TPZ (G*m*PCT) solution with a concentration of 10 mg/mL.

Characterization methods

^1H NMR spectra were recorded using a Bruker AV-400 NMR spectrometer. All samples were dissolved in D_2O before measurements. UV-vis spectra were collected using a Lambda 25 UV-vis spectrophotometer (Perkin Elmer, Waltham, MA). Samples were dispersed in water before measurements. Transmission electron microscopy (TEM) imaging was executed using a JEOL 2100F analytical electron microscope (JEOL, Tokyo, Japan) at an operating voltage of 200 kV. Samples were prepared by dropping an aqueous particle suspension onto a carbon-coated copper grid and air-dried before measurement. Zeta potential and dynamic light scattering (DLS) were tested using a Malvern Zetasizer Nano ZS system (Worcestershire, UK) coupled with a standard laser with a wavelength of 633 nm. Cu content of the synthesized nanocomposite were analyzed using a Leeman Prodigy inductively coupled plasma-optical emission spectroscopy (ICP-OES, Hudson, NH).

Blood compatibility test

All animal experiments were performed in accordance with the guidelines from the Institutional Committee for Animal Care and Use of Donghua university and also with the regulations of the National Ministry of Health. Male BALB/c nude mice (5-6 week-old) were supplied by Shanghai SLAC Laboratory Animal Co., Ltd (Shanghai, China). 2 mL whole blood was taken from the BALB/c nude mice and added into the anticoagulant tube. After centrifugation (3000 rpm, 5min) and washing sediment with PBS for 5 times, the red blood cells were collected and then diluted with PBS for 50 times. 100 uL of the above diluted red blood cells were added to 7 centrifuge tubes containing 900 μL of GPPCT solution ([TPZ]=2.5, 5, 10, 15, 20 uM), ultrapure water or PBS and mixed evenly. After incubation at 37 °C for 2 h, the mixtures were centrifuged at 10000 rpm for 5 min, and the UV absorbance of supernatant of each tube at 540 nm was measured. Hemolysis rate is calculated according to the following formula:

$$\text{Hemolysis rate (\%)}=(D_t - D_{nc}) / (D_{pc} - D_{nc})\times 100\ \%$$

D_t is the light absorption value of the test sample at 540 nm, D_{pc} and D_{nc} are the light absorption value of the positive control and the bright control at 540 mm, respectively.

Drug loading efficiency

A standard absorbance-concentration calibration curve of TPZ was measured and plotted. Then, TPZ encapsulation efficiency and percentage of G*m*PC and GPPC was determined by collecting the free TPZ left in the filtrate after centrifugation through ultrafiltration tubes and measuring its absorbance at 460 nm using a Lambda 25 UV-vis spectrophotometer (PerkinElmer, Waltham, MA). The drug loading content (LC %) and entrapment efficiency (EE %) of nanoplatforms can be calculated according to the following formulas of (1) and (2), where M_t, M_0 and M_L stand for the masses of the encapsulated TPZ, the initial TPZ and the drug loaded nanocomplexes, respectively.

$$LC\ \% = M_t / M_0 \times 100\ \% \quad (1)$$

$$EE\ \% = M_t / M_L \times 100\ \% \quad (2)$$

In *vitro* release profile of TPZ and Cu(II)

The *in vitro* drug release profile of TPZ and Cu(II) from GPPCT was investigated in various buffer solutions of pH 7.4, 6.5 and 5.5. GPPCT were dispersed in 1 mL corresponding buffer solution to the final concentration of 1 mg/mL, and then the solutions were sealed in a dialysis bag with MWCO of 8000~14000. The dialysis bag was immersed in 20 mL buffer solutions of pH 7.4, 6.5 or 5.5 and placed in a shaker at 37 °C for 48 h. Within a specified time interval (0.5, 1, 2, 4, 6, 8, 12, 24, 36, 48 h), 1 mL sample solution was taken out from buffer solution to measure the released amount of TPZ by UV-vis spectra, and an equivalent amount of fresh buffer solution was added. Meanwhile, the concentration of Cu in the solutions at different time points was determined by ICP-OES.

Measurement of ·OH generation

The GPPC, GPPC+GSH, GPPCT, GPPCT+GSH and TPZ, TPZ+GSH solution (with a final

concentration of 200 μM TPZ and 616 μM Cu(II) with the addition of GSH (5 mM or 10 mM) and H_2O_2 solution (with a final concentration of 10 mM) were mixed with the methylene blue (MB) solution (with a final concentration of 10 μg/mL) to reach a final volume of 3 mL. Then the mixtures kept at room temperature for 4 h. The ·OH-induced MB degradation was monitored by the change in the absorbance value at 400-800 nm *via* UV-vis spectra. For comparison, the solution with MB alone, the H_2O_2 and MB mixed solution, GPPC or TPZ and MB mixed solution with the same MB, H_2O_2, GPPC or TPZ concentrations as those of the above samples were also tested in the same way.

After mixing the GPPCT solution with H_2O_2 and MB solution with or without the addition of GSH (10 mM), the absorbance value of the mixture at 665 nm within 10 h was detected by UV-vis, and the influence of the presence of GSH on ROS generation rate of the nanoplatforms was studied. In addition, GPPCT solution with or without the addition of GSH (10 mM) were mixed with H_2O_2 and MB solution under different pH of 5.5, 6.5 and 7.4, respectively, at room temperature for 4 h. The changes of absorbance values in the range of 400-800 nm were detected by UV-vis to study the effect of different pHs on ROS generation rate of the nanoplatforms.

Cell culture

4T1 cells (a mouse breast cancer cell line) and L929 cells (a mouse fibroblast cell line) was obtained from the Institute of Biochemistry and Cell Biology, the Chinese Academy of Sciences (Shanghai, China). 4T1 cells were regularly cultured in DMEM medium containing 10 % FBS and 1 % penicillin-streptomycin and kept at 37 °C in a Thermo Scientific cell incubator (Waltham, MA) with 5 % CO_2.

Hypoxia 4T1 cancer cell model

Hypoxia 4T1 cancer cell model was established through the addition of $CoCl_2$ into the cell medium according to previous reports [3], and the hypoxia condition of cells was verified using a CCK-8 assay. Briefly, 4T1 cells were seeded in two 96-well plates at a density of 1×10^4 cells per well in 100 μL of

DMEM containing different concentrations of $CoCl_2{\cdot}6H_2O$ (10, 20, 30, 50, 100, 150, 200, 250 μM) for 24 h. Then cell viability of one of the plate of cells was detected by a CCK-8 method. Meanwhile, the other plate of cells was further added with 2.5 μM GPPCT per well and cultured for another 24 h to determine the cell viability. After assuring the optimized $CoCl_2{\cdot}6H_2O$ concentration through the above assays, the cells were collected to test the expression of HIF-1α in cells with the addition of $CoCl_2{\cdot}6H_2O$ by western blot, so as to verify whether the hypoxia model was successfully constructed

Cytotoxicity assay

4T1 cells were seeded in a 96-well plate at a density of 1×10^4 cells per well in 100 μL DMEM overnight to bring the cells to confluence (the hypoxia group was simulated with $CoCl_2{\cdot}H_2O$). Then, the cells were washed with pre-warmed sterile PBS for 3 times, and cultured using fresh medium containing different concentrations of as-prepared nanomaterials for 24 h. The medium in each well was replaced with 100 μL of serum-free medium containing 10 % CCK-8 solution and the cells were incubated for another 3 h under regular cell culture conditions. After that, the cells in each well were analyzed using Thermo Scientific Multiskan MK3 ELISA reader (Thermo Scientific, Waltham, MA) at a wavelength of 450 nm. Each sample was tested in six replicates. The cell viability of 4T1 cells and L929 cells under normoxia was tested in a similar way. The half-maximal inhibitory concentration (IC_{50}) of each material was calculated using a Statistical Product and Service Solutions (SPSS) software (International Business Machines Corporation, New York, NY).

Cellular uptake assay of Cu in 4T1 cells

4T1 cells were seeded in a 12-well plate at a density of 10×10^4 cells per well in 1 mL of DMEM medium overnight. Afterward, the medium was replaced with 1 mL of fresh medium containing GPPCT and G*m*PCT ([Cu]=0, 5, 10, 20 μM) and cells were incubated for 24 h. The cells were then rinsed three times with PBS and trypsinized. After counting the cell number in the suspensions, the

cells were centrifuged to remove the supernatant, collected, digested by *aqua regia* and diluted to 5 mL. Finally, ICP-OES was performed to determine the intracellular Cu content of samples.

Cellular uptake and intracellular localization of TPZ in 4T1 cells

4T1 cells were seeded in 6-well plates at a density of 20×10^4 cells per well in 1 mL of DMEM medium (the hypoxia group was simulated with $CoCl_2\cdot H_2O$). After 24 h incubation, the cell medium was replaced with fresh medium containing PBS, GPPCT or G*m*PCT ([TPZ]=2.5 μM) and the cells were incubated for 6 h. Cells treated with PBS were set as blank control. The cells were then rinsed three times with PBS, trypsinized, centrifuged and finally collected in PBS for analysis using a Becton Dickinson FACScan flow cytometer (BD Biosciences, Franklin Lake, NJ).

At the same time, 4T1 cells were seeded in petri dishes at a density of 10×10^4 per dish and received the same treatments as above. Afterward, the cells were lightly washed with PBS buffer solution, fixed with paraformaldehyde for 15 min and stained with DAPI for 30 min. Finally, a confocal laser scanning microscope microscope (CLSM, Zeiss, Jena, LSM700) was used to directly observe intracellular uptake and distribution of TPZ.

Determination of ROS and GSH/GSSG levels in 4T1 cells

For ROS quantification, 4T1 cells were seeded in 6-well plates at a density of 20×10^4 cells per well in 1 mL of DMEM medium overnight (the hypoxia group was simulated with $CoCl_2\cdot H_2O$). Then the cell medium was replaced with serum-free medium containing the GPPC and GPPCT ([Cu]=7.69 μM, the concentration of TPZ is equivalent to 2.5 μM) for 6 h. Cells treated with PBS were used as control. Then the cells were washed with PBS before the addition of 1 mL of serum-free medium containing 10 μM DCFH-DA. After incubation for 20 min at 37 °C in the dark, the cells were washed twice with PBS and were analyzed with CLSM and flow cytometer.

For GSH/GSSG detection, 4T1 cells were seeded in 6-well plates at a density of 20×10^4 cells per well

in 1 mL of DMEM medium (the hypoxia group was simulated with $CoCl_2 \cdot H_2O$). After 24 h incubation, the cell medium was replaced with fresh medium containing PBS, GPPC or GPPCT ([Cu]=7.69 μM, the concentration of TPZ is equivalent to 2.5 μM) and the cells were incubated for 6 h. Cells treated with PBS were used as control. The cells were then rinsed three times with PBS, trypsinized and collected to measure the intracellular GSH content according using GSH and GSSG Assay Kits (Beyotime Biotechnology, Shanghai, China).

Animal models

Male BALB/c nude mice (15~20 g, 5~6 weeks, Shanghai Slac Laboratory Animal Center, Shanghai, China) were xenografted with 4T1 tumors by inoculating 4T1 cells (250×10^4 cells, 100 μL cell suspension) subcutaneously into the left rear of the mice (root of the thigh). After being reared in animal house for two weeks (the tumor volume reached 150~350 mm^3), the tumor models were successfully constructed for the treatment with different reagents.

***In vivo* antitumor activity against a subcutaneous xenograft 4T1 tumor model**

The 4T1 xenograft tumor-bearing BALB/c nude mice were randomly divided into 6 groups (5 for each group): PBS group, GPP group, GPPC group, CPPCT group, G*m*PCT group and TPZ group (The concentration of the materials was prepared with sterile PBS, [TPZ]=2 mg/kg, 100μL), and it was injected into tumor-bearing mice through tail vein once every three days. The tumor volumes and the body weights were recorded every third day. The tumor volume was calculated according to a formula of $V=W^2\times L/2$, where W and L represent the width and length of tumor, respectively. The relative tumor volume was calculated based on the tumor volume of the first day. After 20 days, the tumor tissues and the main organs (heart, liver, spleen, lung, and kidney) were removed from the sacrificed mice for H&E, TUNEL, Ki-67, HIF-1α and DHE staining according to standard protocols [4, 5].

***In vivo* biodistribution**

The 4T1 tumor-bearing mice were intravenously injected with the GPPCT or G*m*PCT complexes ([Cu(II)]=6.9 mM, in 100 μL PBS for each mouse). After the mice were sacrificed at the given time points (2, 8, 24, 48, or 72 h), their heart, liver, spleen, lung, kidney and tumor were extracted and weighed. The weighed organs and tumors were then digested with 2 mL aqua regia (about 7 days) and diluted with twice the volume of ultrapure water. Finally, the Cu content in these samples was measured by ICP-OES and the data were expressed as mean ± SD (n=3).

Blood hematology and biochemistry analysis

At 20 days post-treatment, blood samples were harvested from the mice in each group. The collected blood solutions (~ 500 μL) were treated with anticoagulant (EDTA) for hematology analysis. Another 500 μL of residual blood was collected from each mouse, set at 4 °C for 2 h, and centrifuged at 2000 rpm to obtain the blood serum, which was used for biochemistry analysis. Blood hematology and biochemistry test indicators such as white blood cells (WBC), red blood cells (RBC), hemoglobin (HGB), means corpuscular hemoglobin (MCH), lymphocytes percentage (LYM), hematocrit (HCT), means corpuscular volume (MCV), means corpuscular hemoglobin concentration (MCHC), platelets (PLT), monocyte (MON), granulocyte (GRAN) and alanine aminotransferase (ALT), aspartate aminotransferase (AST), urea (UREA), uric acid (UA), creatinine (CREA) were obtained at Servicebio (Wuhan, China).

Statistical analysis

Data were presented as the means ± standard deviations. One-way analysis of variance statistical analysis was adopted to analyze the significance of the experimental data. A p value of 0.05 was selected as the significance level, and the data were marked with (ns) for $p > 0.05$, (*) for $p < 0.05$, (**) for $p < 0.01$, and (***) for $p < 0.001$, respectively.

Additional Table and Figures

Table S1. The TPZ drug loading content (LC %) and entrapment efficiency (EE %) of GPPCT and G*m*PCT.

Sample	LC (%)	EE (%)
GPPCT	2.26	65.03
G*m*PCT	2.38	66.67

Table S2. The hydrodynamic diameters, polydispersity index and Zeta potential of GPP, G*m*P, GPPC, G*m*PC, GPPCT and G*m*PCT.

Sample	Hydrodynamic diameters (nm)	Polydispersity index (PDI)	ζ-potential (mV)
GPP	187.2 ± 9.8	0.620 ± 0.087	7.0 ± 0.2
G*m*P	193.6 ± 10.0	0.448 ± 0.051	7.6 ± 0.5
GPPC	171.8 ± 13.8	0.667 ± 0.133	14.4 ± 7.7
G*m*PC	175.6 ± 14.3	0.590 ± 0.186	15.2 ± 1.3
GPPCT	150.3 ± 3.1	0.270 ± 0.022	20.3 ± 4.0
G*m*PCT	151.4 ± 5.2	0.292 ± 0.078	21.5 ± 5.9

Table S3. IC_{50} value and safety index of GPPCT for different cells.

Cells	IC_{50} (μM)	Safety index IC_{50}L929/ IC_{50}4T1
4T1 (hypoxia)	5.032	4.780
4T1 (normoxia)	11.70	2.056
L929 (normoxia)	24.054	——

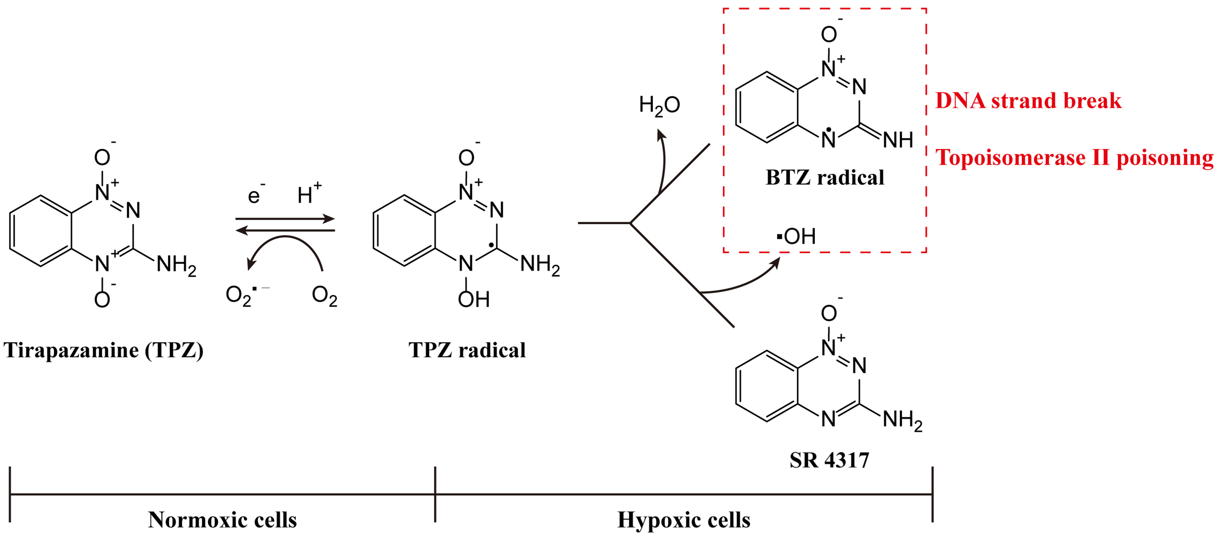

Fig. S1 The action mechanism of TPZ under normal and hypoxic conditions.

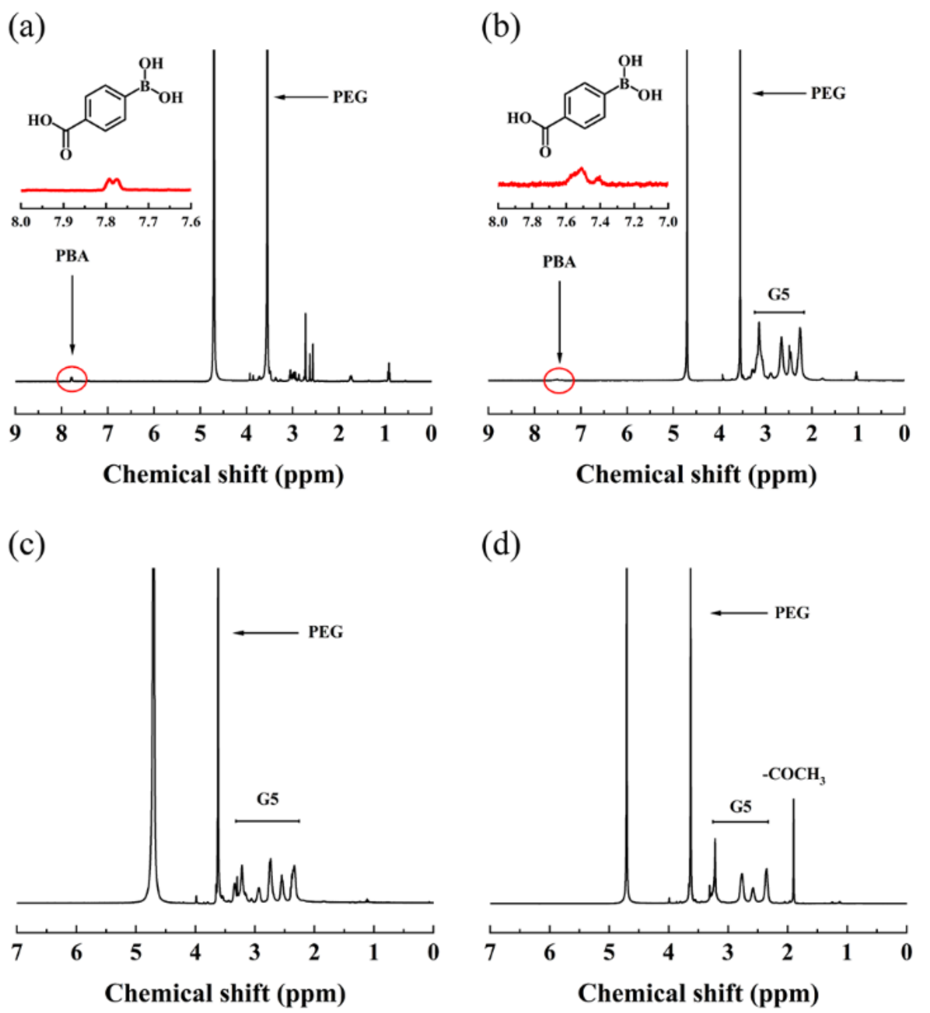

Fig. S2 ^{1}H NMR spectra of (a) PBA-PEG-COOH, (b) G5.NH$_2$-PEG-PBA, (c) G5.NH$_2$-*m*PEG and (d) G5.NHAc-*m*PEG.

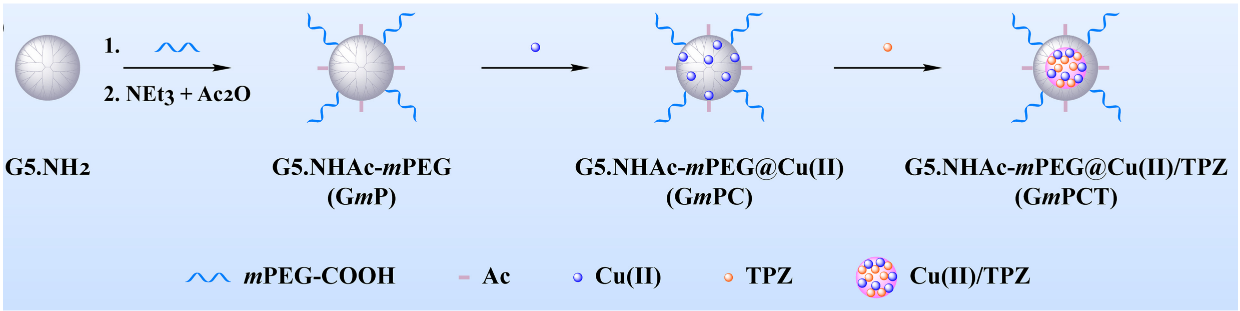

Fig. S3 Schematic diagram of the synthesis of G5.NHAc-*m*PEG@Cu(II)/TPZ (G*m*PCT).

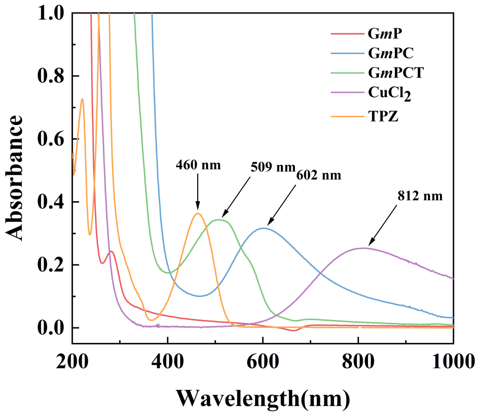

Fig. S4 The UV-vis spectra of G*m*P, G*m*PC, G*m*PCT, $CuCl_2$ and TPZ.

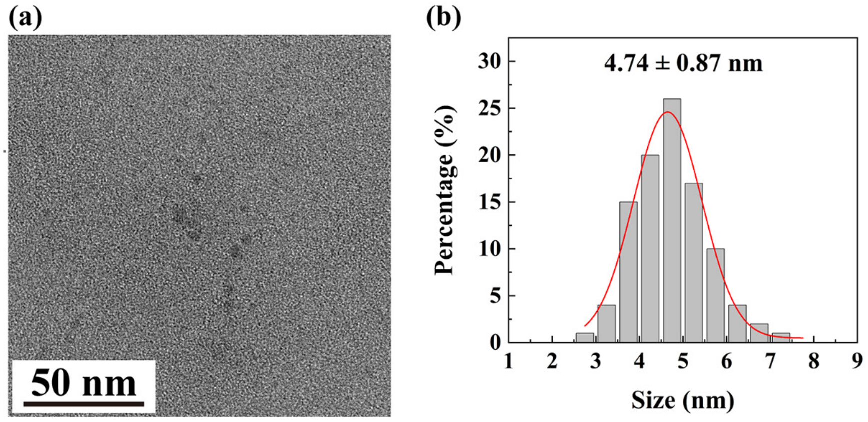

Fig. S5 (a) TEM image and (b) size distribution histograms of G*m*PCT.

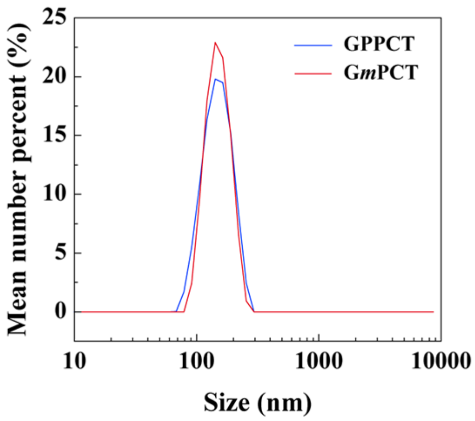

Fig. S6 Hydrodynamic diameter histogram of GPPCT and G*m*PCT.

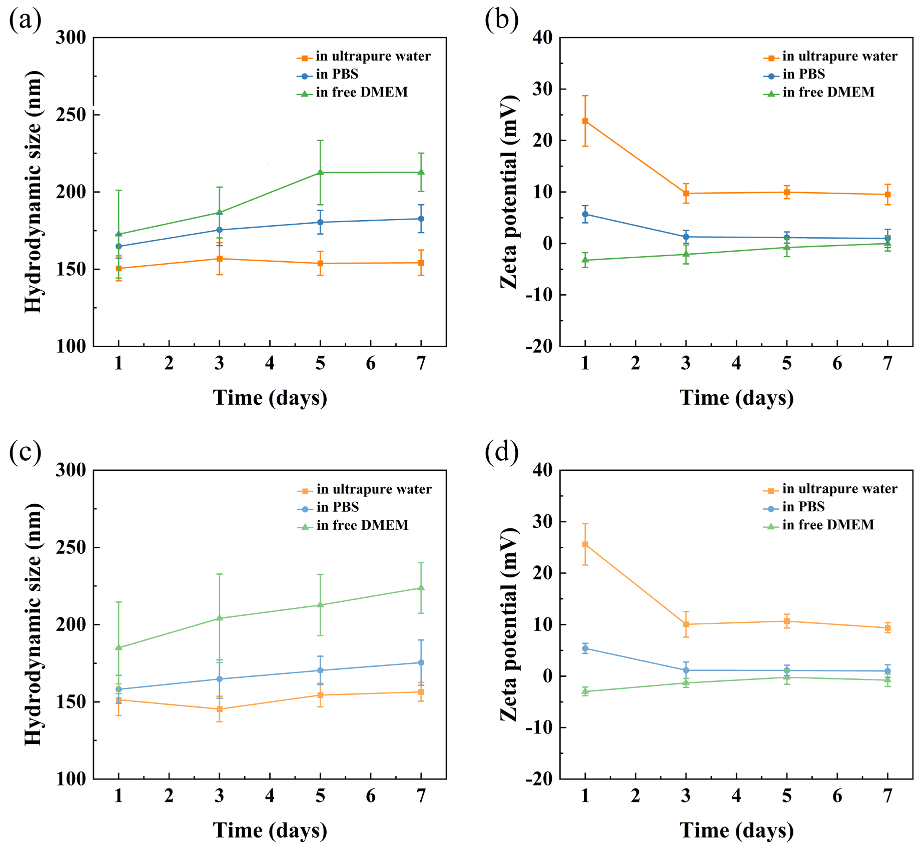

Fig. S7 Changes of hydrodynamic diameters and Zeta potential of (a, b) GPPCT and (c, d) G*m*PCT in water, PBS and DMEM medium for 1, 3, 5 and 7 days.

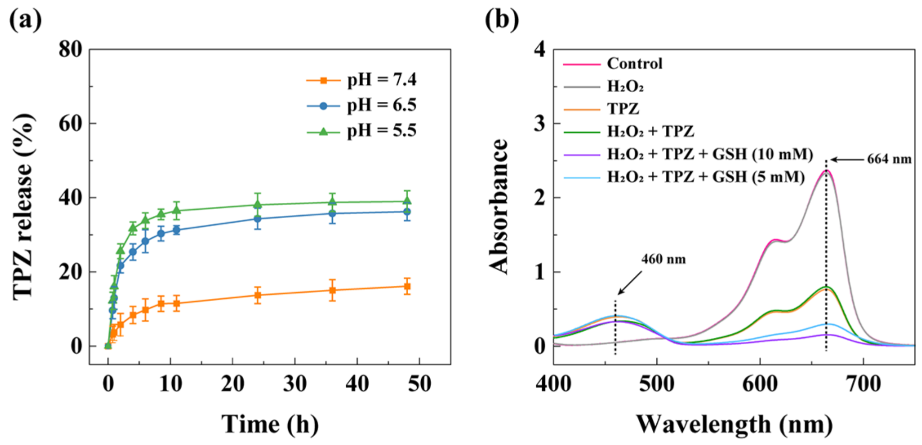

Fig. S8 (a) Accumulative release of TPZ from GPPCT in buffer solutions with different pH of 5.5, 6.5 and 7.4 at 37 °C ([GPPCT]=1 mg/mL). (b) UV-vis spectra of MB treated with H_2O_2 and TPZ with or without the presence of GSH for 4 h ([TPZ]=200 μM).

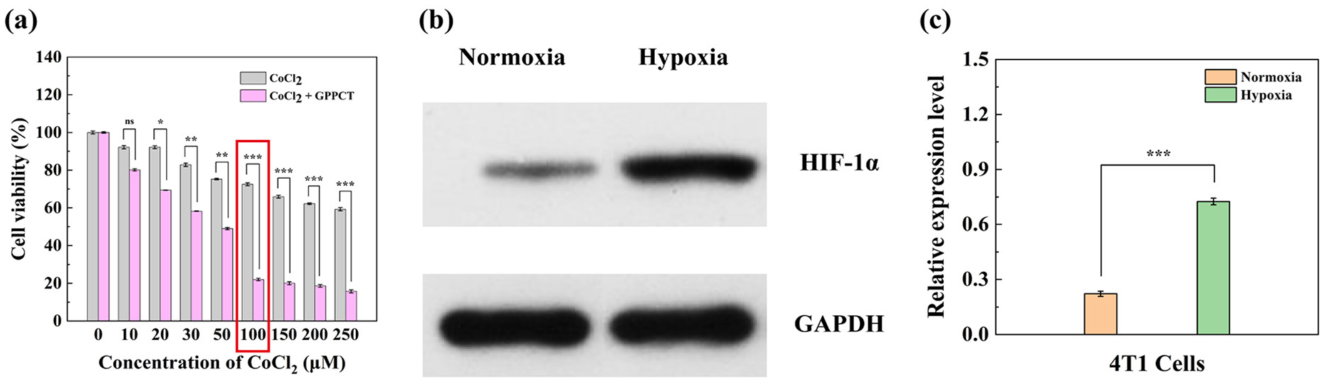

Fig. S9 (a) CCK-8 assay of 4T1 cells under different concentrations of $CoCl_2 \cdot H_2O$ in the presence or absence of GPPCT ([TPZ]=2.5 μM). (b) HIF-1α expression of 4T1 cells in the presence or absence of $CoCl_2 \cdot H_2O$ determined by western blot and (c) its quantitative analysis results ([$CoCl_2 \cdot H_2O$]=100 μM).

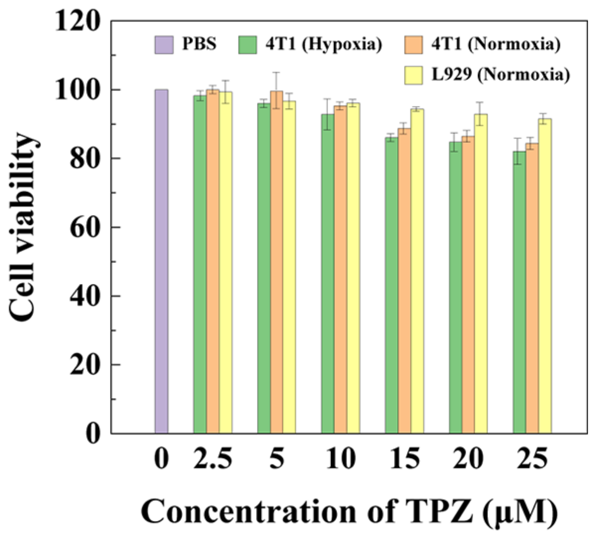

Fig. S10 Cell viability of 4T1 (hypoxia and normoxia) and L929 (normoxia) cells after co-culture with GPP detected by CCK-8 assay.

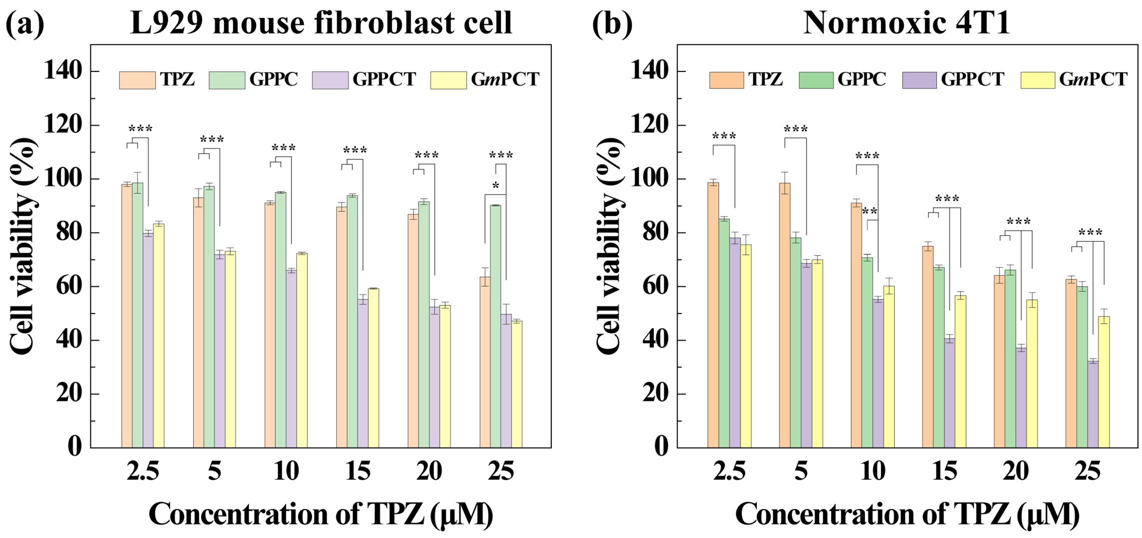

Fig. S11 Cell viability of (a) L929 cells and (b) normoxic 4T1 cells after co-culture with free TPZ, GPPC, GPPCT and G*m*PCT for 24 h detected by CCK-8 assay.

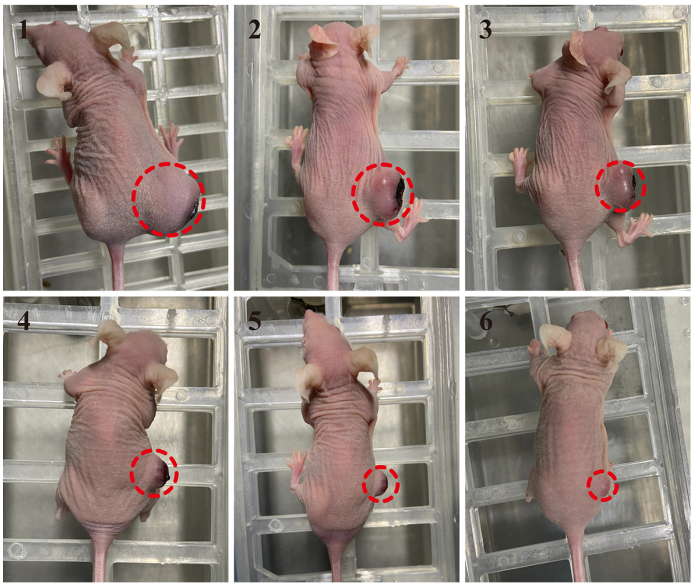

Fig. S12 Representative images of tumor bearing mice with different treatments on the 20th days post-treatment. (1: PBS, 2: GPP, 3: TPZ, 4: GPPC, 5: G*m*PCT and 6: GPPCT.)

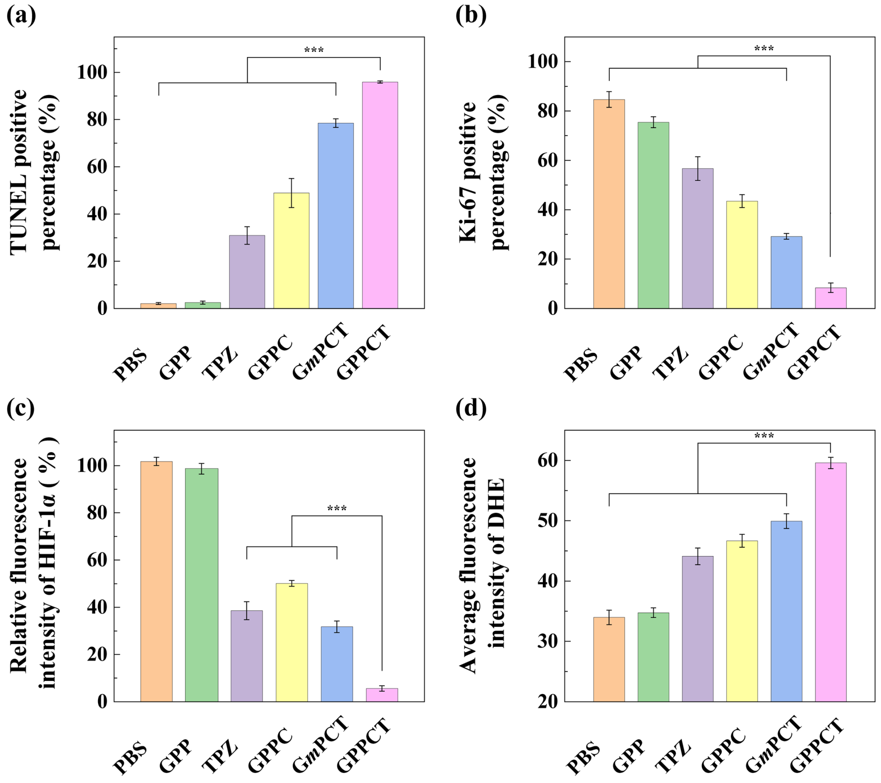

Fig. S13 Quantification of (a) TUNEL mean fluorescence intensity, (b) positive signals of Ki-67, (c) HIF-1α and (d) DHE mean fluorescence intensity after different treatments. *** $p < 0.001$.

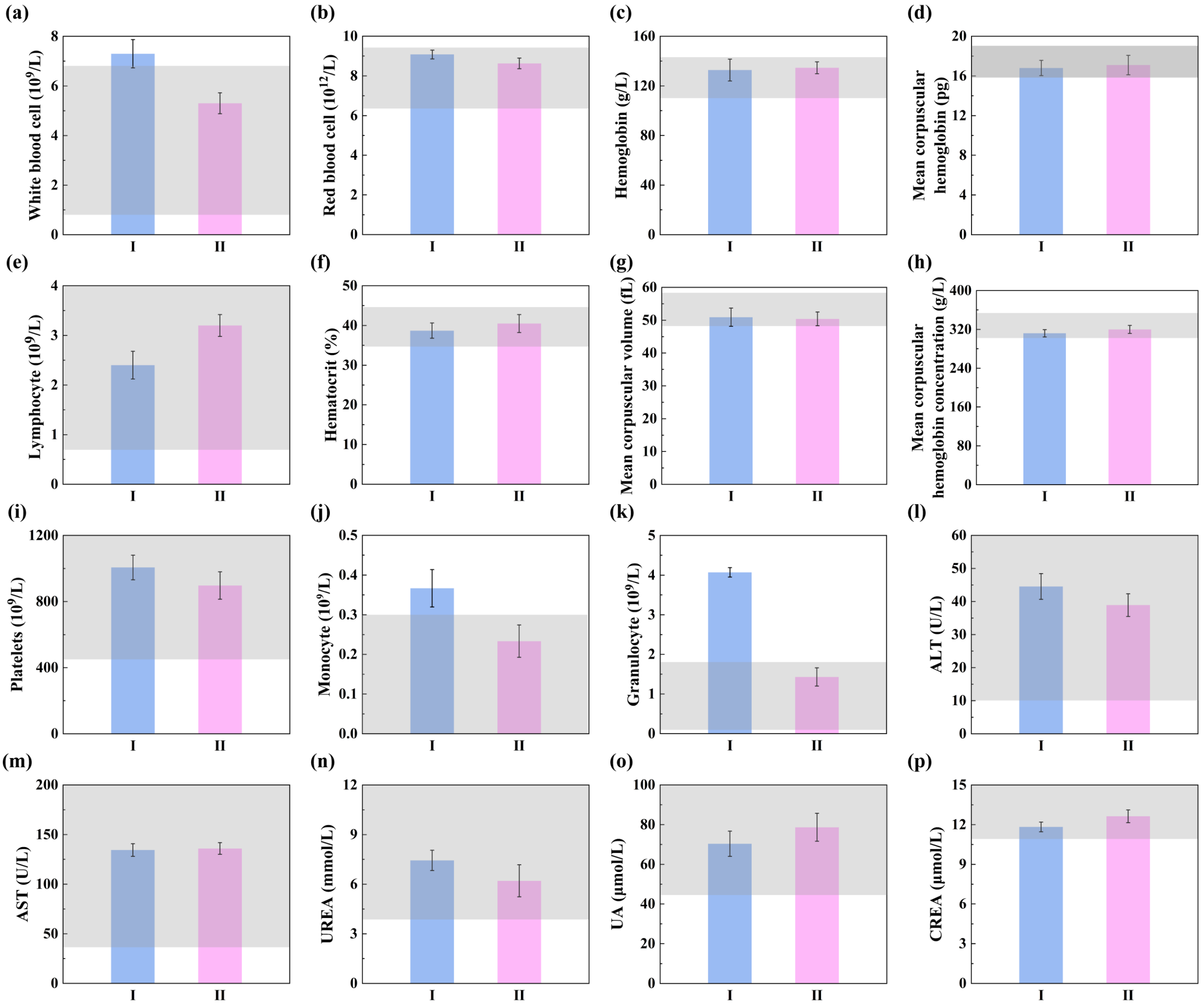

Fig. S14 Blood biochemistry analysis of (a) WBC, (b) RBC, (c) HGB, (d) MCH, (e) LYM, (f) HCT, (g) MCV, (h) MCHC, (i) PTL, (j) MON and (k) GRAN of mice on the last day of different treatments of (I) PBS and (II) GPPCT. Liver function index of (l) ALT and (m) AST and kidney function index of (n) UREA, (o) UA and (p) CREA of mice on the last day of with different treatments of (I) PBS and (II) GPPCT (n=3).

References

1. Liu J, Xiong Z, Zhang J, Peng C, Klajnert-Maculewicz B, Shen M, Shi X. Zwitterionic gadolinium(III)-complexed dendrimer-entrapped gold nanoparticles for enhanced computed tomography/magnetic resonance imaging of lung cancer metastasis. ACS Applied Materials & Interfaces. 2019;11**:**15212-15221.
2. Hou WX, Wei P, Kong LD, Guo R, Wang SG, Shi XY. Partially PEGylated dendrimer-entrapped gold nanoparticles: a promising nanoplatform for highly efficient DNA and siRNA delivery. J Mater Chem B. 2016;4**:**2933-2943.
3. Lee HR, Leslie F, Azarin SM. A facile in vitro platform to study cancer cell dormancy under hypoxic microenvironments using $CoCl_2$. Journal of Biological Engineering. 2018;12.
4. Ouyang ZJ, Li D, Xiong ZJ, Song C, Gao Y, Liu RN, Shen MW, Shi XY. Antifouling dendrimer-entrapped copper sulfide nanoparticles enable photoacoustic imaging-guided targeted combination therapy of tumors and tumor metastasis. ACS Appl Mater Interfaces. 2021;13**:**6069-6080.
5. Yang C, Gao Y, Fan Y, Cao L, Li J, Ge YL, Tu WZ, Liu Y, Cao XY, Shi XY. Dual-mode endogenous and exogenous sensitization of tumor radiotherapy through antifouling dendrimer-entrapped gold nanoparticles. Theranostics. 2021;11**:**1721-1731.
